# Pretreatment neutrophil-to-lymphocyte ratio and mutational burden as biomarkers of tumor response to immune checkpoint inhibitors

**DOI:** 10.1038/s41467-021-20935-9

**Published:** 2021-02-01

**Authors:** Cristina Valero, Mark Lee, Douglas Hoen, Kate Weiss, Daniel W. Kelly, Prasad S. Adusumilli, Paul K. Paik, George Plitas, Marc Ladanyi, Michael A. Postow, Charlotte E. Ariyan, Alexander N. Shoushtari, Vinod P. Balachandran, A. Ari Hakimi, Aimee M. Crago, Kara C. Long Roche, J. Joshua Smith, Ian Ganly, Richard J. Wong, Snehal G. Patel, Jatin P. Shah, Nancy Y. Lee, Nadeem Riaz, Jingming Wang, Ahmet Zehir, Michael F. Berger, Timothy A. Chan, Venkatraman E. Seshan, Luc G. T. Morris

**Affiliations:** 1 Department of Surgery, Memorial Sloan Kettering Cancer Center, New York, NY USA; 2 Immunogenomics and Precision Oncology Platform, Memorial Sloan Kettering Cancer Center, New York, NY USA; 3 Human Oncology and Pathogenesis Program, Memorial Sloan Kettering Cancer Center, New York, NY USA; 4 Information Systems, Memorial Sloan Kettering Cancer Center, New York, NY USA; 5 Department of Medicine, Memorial Sloan Kettering Cancer Center, New York, NY USA; 6 Department of Pathology, Memorial Sloan Kettering Cancer Center, New York, NY USA; 7 Department of Radiation Oncology, Memorial Sloan Kettering Cancer Center, New York, NY USA; 8 Department of Epidemiology and Biostatistics, Memorial Sloan Kettering Cancer Center, New York, NY USA

**Keywords:** Biomarkers, Translational research, Cancer genetics, Cancer immunotherapy, Tumour immunology

## Abstract

Treatment with immune checkpoint inhibitors (ICI) has demonstrated clinical benefit for a wide range of cancer types. Because only a subset of patients experience clinical benefit, there is a strong need for biomarkers that are easily accessible across diverse practice settings. Here, in a retrospective cohort study of 1714 patients with 16 different cancer types treated with ICI, we show that higher neutrophil-to-lymphocyte ratio (NLR) is significantly associated with poorer overall and progression-free survival, and lower rates of response and clinical benefit, after ICI therapy across multiple cancer types. Combining NLR with tumor mutational burden (TMB), the probability of benefit from ICI is significantly higher (OR = 3.22; 95% CI, 2.26-4.58; *P* < 0.001) in the NLR low/TMB high group compared to the NLR high/TMB low group. NLR is a suitable candidate for a cost-effective and widely accessible biomarker, and can be combined with TMB for additional predictive capacity.

## Abstract (web summary)

There is an unmet clinical need for simple, accessible biomarkers to select patients who are more likely to respond to immune checkpoint therapy. Here the authors show that a lower neutrophil-to-lymphocyte ratio is associated with better overall and progressive-free survival, as well as higher rate of response, in a multi-cancer cohort of patients treated with immune checkpoint inhibitors.

## Introduction

In recent years, immune checkpoint inhibitors (ICIs) targeting inhibitory receptors on T cells have achieved tumor responses in a subset of patients across a wide range of cancer types. However, most patients treated with ICI do not experience clinical benefit. This situation creates a need for biomarkers that would help clinicians identify those patients who are more likely to have extended survival and whose tumors are more likely to respond to ICI. This may help facilitate precise, cost-effective treatments with fewer adverse events.

There are several molecular and genomic biomarkers with predictive value for ICI across multiple cancer types. These include programmed death ligand 1 (PD-L1) expression, tumor mutational burden (TMB), mutations in specific genes, human leukocyte antigen class I zygosity and diversity, and microsatellite instability (MSI) status^1–7^. Within the tumor immune microenvironment, a higher degree of infiltration of myeloid cells and a lower level of infiltration of lymphocytes have each been associated with worse outcomes, including in patients treated with ICI^8–11^. However, these biomarkers have limitations that have precluded widespread clinical use, such as the need for sufficient tumor tissue, the need to sequence DNA extracted from tumor, and the lack of standardized quantitative scoring systems for immune cell or PD-L1 immunohistochemistry^12,13^. There is a clinical need for predictive biomarkers that can be easily obtained at low cost, in diverse (including resource-poor) settings, and without recourse to advanced genomic technologies or specialized histopathologic expertise. Candidates for cost-effective and accessible biomarkers include factors in peripheral blood.

In the context of cancer, work on immune-related markers in peripheral blood has focused on the neutrophil-to-lymphocyte ratio (NLR). This ratio, defined by the absolute counts of neutrophils and lymphocytes, may represent the balance between pro-tumoral inflammatory status and anti-tumoral immune response. An extensive body of research describes NLR as a general prognostic factor across several cancer types^14–20^. It is unknown, however, whether NLR is associated with prognosis in patients treated with ICI or has predictive value for the likelihood of response to ICI. Most studies examining this question have been performed either in small subsets of patients or in specific cancer types. The association of NLR with ICI response is not well understood^21–29^.

The aim of this retrospective cohort study is to analyze the association of pretreatment peripheral blood NLR with survival and response rates, in a large series of patients diagnosed with a wide range of cancer types treated with ICI. Secondarily, we analyze the predictive value of combining this clinical marker of host immunity (NLR) with a genomic marker of tumor antigenicity (TMB), by integrating tumor sequencing data.

In this work, we show that higher NLR is associated with poorer survival and a lower probability of response to immunotherapy, both at a pan-cancer level and within several cancer types. Moreover, combining NLR with TMB provides additional predictive value in patients treated with ICI.

## Results

### Pan-cancer analysis of NLR

To assess the predictive value of pretreatment NLR, we analyzed data for 1714 patients with response and survival outcomes after ICI treatment (Supplementary Fig. 1, Supplementary Table 1, and Supplementary Data 1). Median age was 64 years (interquartile range (IQR) 55–71); 926 patients (54%) were male. The median follow-up time was 18 months (IQR 12–26). The cohort had an overall response rate (defined as partial or complete response) of 27% and an overall clinical benefit defined as reponse or stable disease for for ≥6 months) rate of 32%.

**Fig. 1:**
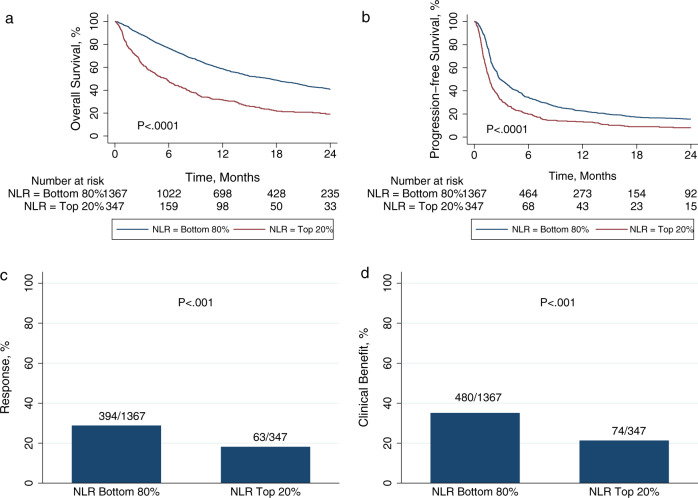
Pan-cancer outcomes based on neutrophil-to-lymphocyte ratio (NLR). **a** Overall survival, **b** progression-free survival, **c** response, and **d** clinical benefit at a pan-cancer level based on high vs. low NLR. The cutoff was the top 20th percentile within each cancer type. *P*-values are according to log-rank test for **a** and **b**, and Pearson’s *χ*^2^-test for **c** and **d**.

As NLR distribution in different cancer types was similar but not identical (Supplementary Fig. 2), we analyzed NLR values by percentile within cancer type. Patients with NLR values in the top 20th percentile within cancer type had significantly poorer overall survival (OS) (hazard ratio (HR) = 2.17; 95% confidence interval (95% CI) 1.89–2.50; *P* < 0.001) and progression-free survival (PFS) (HR = 1.60; 95% Cl 1.41-1.81;*P* < 0.001), and poorer rates of response (18% vs. 29%; *P* < 0.001) and clinical benefit (21% vs. 35%; *P* < 0.001) (Fig. 1). These differences were highly robust across the range of decile cutoffs (Supplementary Figs. 3–6). Higher NLR was associated with significantly poorer OS for all decile cutoffs from 10th to 90th percentile, significantly poorer PFS for cutoffs from 10th to 80th percentile, significantly lower rate of clinical benefit from 10th to 80th percentile, and significantly lower response rate from 10th to 50th percentile.

### Outcomes stratified by cancer type

**Fig. 2:**
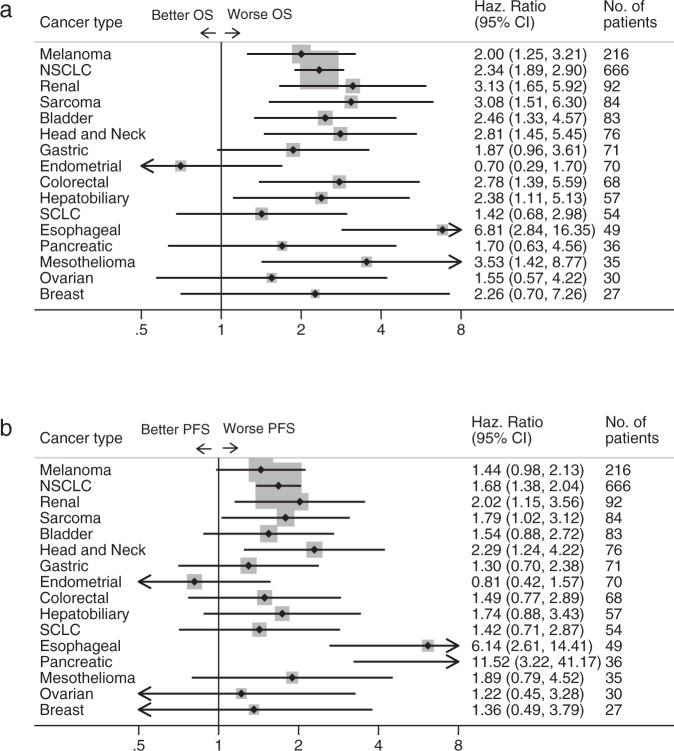
Outcomes based on neutrophil-to-lymphocyte ratio (NLR) stratified by cancer type. Forest plots showing hazard ratios (HRs) with 95% CIs for **a** overall survival and **b** progression-free survival across cancer types, comparing top 20th NLR percentile within each cancer type to bottom 80th. HRs were calculated with Cox proportional hazard regression. Abreviations: OS, overall survival; Haz. Ratio, hazard ratio; CI, confidence interval; NSCLC, non-small cell lung cancer; SCLC, small cell lung cancer; PFS, progression-free survival.

To examine the consistency of these trends across cancer types, we performed an analysis within each cancer, considering NLR values within the top 20th percentile of that cancer type as high NLR. This analysis confirmed that high NLR was associated with poorer OS and PFS (Fig. 2). Although the HRs of survival were directionally consistent across nearly all cancer types, statistical significance was not observed in some cancer types, in particular those with smaller cohort size. Similar results were found for response and clinical benefit; patients with high NLR had a lower probability of response or clinical benefit from ICI across most cancer types, although the results were not statistically significant for all cancers (Supplementary Fig. 7).

We then performed an exploratory analysis using a prespecified universal cutoff for all cancer types. We selected an NLR value of 5, which has been widely used in the literature across multiple cancer types. We observed similar results with this pan-cancer cutoff (Supplementary Figs. 8 and 9), although we caution that further data are needed to define validated optimal cutoffs for each cancer type.

### Validation of NLR as predictive factor in an independent cohort

To validate these findings, we next assessed the predictive value of high NLR in an independent cohort. We identified 323 additional patients treated with ICI at our center (Supplementary Table 2 and Supplementary Data 1). In this validation cohort, using the same definitions of high NLR (top 20% within cancer type), we observed similar results, with high NLR associated with significantly poorer OS (HR = 1.83; 95% CI 1.33–2.52; *P* < 0.001) and PFS (HR = 1.63; 95% CI 1.22–2.18; *P* = 0.001), and a lower rate of response (17% vs. 28%; *P* = 0.0052) and clinical benefit (26% vs. 41%; *P* = 0.018; Supplementary Fig. 10). In multivariable analysis, higher NLR  as a continuous variable was significantly associated (OR = 0.92 per unit increase in NLR; 95% CI 0.85-0.99;*P* = 0.035) with the likelihood of clinical benefit from ICI therapy (Supplementary Table 3).

### Independent predictive value of NLR and TMB

We then asked whether another pan-cancer biomarker of ICI response, which could potentially be widely available to clinicians, added value. We examined TMB derived from next-generation sequencing (Supplementary Data 1). TMB had an association with the probability of clinical benefit, but with the opposite directionality from NLR. As TMB value or percentile increased, the probability of clinical benefit increased (Supplementary Fig. 11 and Supplementary Tables 4 and 5).

NLR and TMB were very weakly correlated with one another (Spearman’s correlation coefficient = 0.048; *P* = 0.05; Supplementary Fig. 12), suggesting they may each provide independent predictive value. We then performed multivariable analyses for factors associated with clinical benefit using logistic regression models incorporating patient age, cancer type, performance status, ICI line of treatment, cancer stage, and year of treatment as covariates. NLR (OR = 0.93; 95% CI 0.91–0.96; *P* < 0.001) and TMB (OR = 1.03; 95% CI 1.02–1.04; *P* < 0.001) as continuous variables each independently predicted the likelihood of clinical benefit (Supplementary Table 4). Similar results were obtained when patients were analyzed by high (top 20th percentile) vs. low values (for NLR, OR = 0.55; 95% CI 0.40–0.77; *P* < 0.001; for TMB, OR = 3.16; 95% CI 2.37–4.20; *P* < 0.001) (Supplementary Table 5).

We performed additional analyses to show the degree of discrimination of these two biomarkers (NLR and TMB). The results of the receiver operating characteristic (ROC) and area under the curve (AUC) analyses of NLR and TMB (expressed as percentile within cancer type) to separate patients experiencing clinical benefit vs. no clinical benefit are shown in Supplementary Fig. 13. These data indicate that, although NLR and TMB are both statistically strongly associated with the probability of clinical benefit, the ability of any one single numerical cutoff of NLR or TMB to separate clinical outcome is more modest.

### Predictive capacity of a combined NLR–TMB variable

**Fig. 3:**
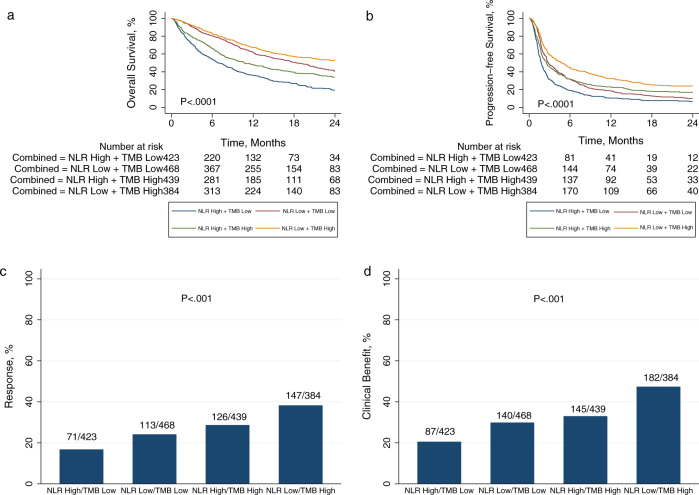
Pan-cancer outcomes according to a combined neutrophil-to-lymphocyte ratio (NLR) and tumor mutational burden (TMB) categorization. **a** Overall survival, **b** progression-free survival, **c** response, and **d** clinical benefit at a pan-cancer level. Patients were classified into four categories based on NLR low/high and TMB low/high, using median within cancer type as cutoff for both variables. *P*-values are according to log-rank test for **a** and **b**, and Pearson’s *χ*^2^-test for **c** and **d**.

**Fig. 4:**
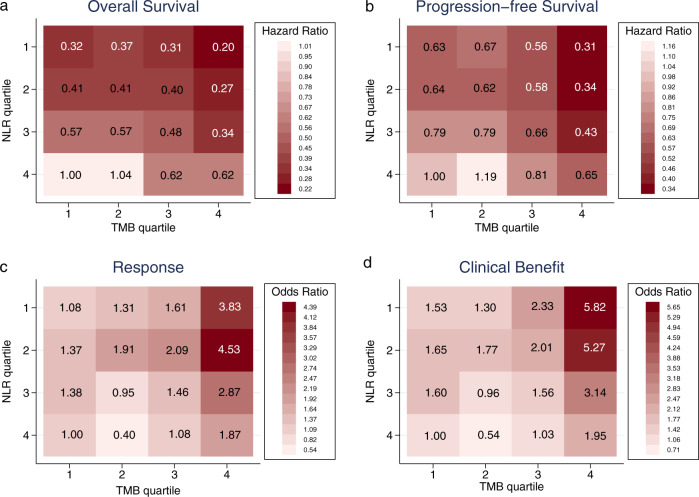
Outcomes across quartile-based categories of neutrophil-to-lymphocyte ratio (NLR) and tumor mutational burden (TMB). Heat maps of hazard ratios (HRs) for **a** overall survival and **b** progression-free survival, and heat maps of odds ratios (ORs) for **c** response and **d** clinical benefit based on 16 within cancer type quartile-based categories of NLR and TMB. HRs were calculated with Cox proportional hazard regression. ORs were calculated with logistic regression.

We assigned patients into four categories based on NLR-low/high and TMB-low/high, using median within cancer type as cutoff for both variables, to divide patients into four equally sized groups. The category with poorest outcomes was NLR-high/TMB-low. Conversely, best outcomes were observed in the NLR-low/TMB-high category. Pan-cancer, the combined NLR–TMB variable stratified patients for all primary endpoints (OS, PFS, response, and clinical benefit) (Fig. 3). The HRs for OS and PFS, and odds ratios (ORs) for response and clinical benefit, are shown for 16 quartile-based categories of NLR and TMB in Fig. 4. Each factor—NLR and TMB—was associated with survival, response, or clinical benefit to a similar degree. Moving from best (NLR-low/TMB-high) to worst (NLR-high/TMB-low) categories, we observed a fivefold change in hazard of OS and odds of clinical benefit, and a threefold change in hazard of PFS and odds of response.

To further assess combining NLR and TMB, we repeated the multivariable analysis of clinical benefit using the combined NLR–TMB variable. The combined categorization not only maintained independent predictive capacity but improved the stratification of patients, with three times higher chance of benefiting from ICI in the NLR-low/TMB-high group compared to the NLR-high/TMB-low group (OR = 3.22; 95% CI 2.26–4.58; *P* < 0.001) (Table 1). Multivariable analyses of OS, PFS, and response showed similar results (Supplementary Tables 6–8).

**Table 1:** Factors associated with clinical benefit from treatment with immune checkpoint inhibitors (ICI).

	Univariate analysis		Multivariable analysis	
	OR (95% CI)	*P*	OR (95% CI)	*P*
NLR + TMB ^a^				
NLR high + TMB low	Ref.		Ref.	
NLR low + TMB low	1.648 (1.212–2.243)	0.001	1.467 (1.032–2.084)	0.03
NLR high + TMB high	1.905 (1.399–2.593)	<0.001	1.858 (1.314–2.628)	<0.001
NLR low + TMB high	3.480 (2.554–4.741)	<0.001	3.220 (2.263–4.581)	<0.001
Age, years	1.014 (1.006–1.022)	<0.001	1.014 (1.004–1.024)	0.007
Sex				
Female	Ref.			
Male	1.146 (0.935–1.405)	0.19		
Cancer type				
Melanoma	Ref		Ref	
NSCLC	0.511 (0.374–0.698)	<0.001	0.968 (0.648–1.446)	0.87
Renal	0.888 (0.544–1.449)	0.63	2.016 (1.114–3.649)	0.020
Sarcoma	0.309 (0.174–0.550)	<0.001	0.885 (0.434–1.806)	0.74
Bladder	0.381 (0.219–0.665)	0.001	0.754 (0.395–1.439)	0.39
Head and Neck	0.328 (0.181–0.593)	<0.001	1.058 (0.532–2.104)	0.87
Gastric	0.611 (0.352–1.060)	0.08	1.455 (0.763–2.776)	0.26
Endometrial	0.664 (0.383–1.151)	0.14	1.804 (0.937–3.474)	0.08
Colorectal	0.352 (0.191–0.649)	0.001	1.033 (0.496–2.148)	0.93
Hepatobiliary	0.312 (0.159–0.613)	0.001	0.766 (0.350–1.676)	0.50
SCLC	0.270 (0.132–0.552)	<0.001	0.745 (0.326–1.703)	0.49
Esophageal	0.670 (0.355–1.262)	0.22	1.904 (0.920–3.940)	0.08
Pancreatic	0.030 (0.004–0.224)	0.001	0.090 (0.012–0.691)	0.021
Mesothelioma	0.313 (0.136–0.720)	0.006	0.825 (0.330–2.061)	0.68
Ovarian	0.211 (0.078–0.573)	0.002	0.641 (0.218–1.887)	0.42
Breast	0.184 (0.062–0.549)	0.002	0.555 (0.150–2.060)	0.38
Performance status				
ECOG 0	Ref		Ref	
ECOG 1	0.519 (0.416–0.647)	<0.001	0.626 (0.484–0.810)	<0.001
ECOG ≥ 2	0.203 (0.121–0.343)	<0.001	0.238 (0.136–0.417)	<0.001
Drug class				
PD-1/PD-L1	Ref			
CTLA-4	0.362 (0.043–3.019)	0.35		
Combo	1.268 (0.973–1.654)	0.08		
ICI line of treatment				
First line	Ref		Ref	
Subsequent line	0.296 (0.238–0.367)	<0.001	0.324 (0.243–0.433)	<0.001
Stage				
I–III	Ref		Ref	
IV	0.480 (0.323–0.714)	<0.001	0.708 (0.444–1.126)	0.15
Year of treatment				
2015–2016	Ref		Ref	
2017–2018	0.792 (0.631–0.993)	0.043	0.763 (0.583–1.000)	0.050

*CI* confidence interval, *Combo* combination of anti-PD-1/PD-L1 and anti-CTLA-4, *CTLA-4* cytotoxic T-lymphocyte antigen 4, *ECOG* Eastern Cooperative Oncology Group, *NLR* neutrophil-to-lymphocyte ratio, *NSCLC* non-small cell lung cancer, *OR* odds ratio, *PD-1* programmed cell death 1, *PD-L1* programmed cell death ligand 1, *SCLC* small cell lung cancer, *TMB* tumor mutational burden.

^a^NLR and TMB were stratified into high and low using median within cancer type as cutoff. ORs were calculated with logistic regression.

Analyzing the main cancer types individually, we found similar results: the NLR-high/TMB-low group had relatively poorer outcomes and the NLR-low/TMB-high group had better outcomes for each of the primary endpoints (OS, PFS, response, and clinical benefit) (Supplementary Figs. 14–17).

To analyze if including early or unknown stage tumors could have biased our results, we performed an additional analysis excluding patients with stage I–II (*n* = 11) or unknown stage (*n* = 32) tumors. Compared to the analysis performed in the entire cohort (Table 1), the results were similar for only stage III–IV tumors (Supplementary Table 9).

MSI-high tumors have generally better prognosis and higher response rates when treated with ICI^5,30–33^. In our cohort, only 57 patients (3%) had an MSI-high tumor. To analyze if MSI status could have affected our results, we performed an additional analysis excluding MSI-high (*n* = 57) or MSI status unknown (*n* = 299) tumors and observed no change in results (Supplementary Table 10).

## Discussion

Chronic inflammation and evasion of immune surveillance are well-established cancer hallmarks^34^. NLR may serve as a surrogate marker of both inflammation status and adaptive immune surveillance, providing a measure of the balance between these two forces. NLR at diagnosis has been described as a prognostic factor, reflecting survival outcome regardless of therapy, across multiple cancer types. However, the predictive value of NLR, reflecting its effect on patient survival and response to a certain therapy, has not been well defined for immunotherapy.

In this study, we found that higher NLR was associated with poorer survival and poorer response rates in a cohort of 1714 cancer patients treated with ICI and validated these findings in an additional cohort of 323 patients. We observed statistically significant associations for OS, PFS, response, and clinical benefit, suggesting that NLR has prognostic and predictive value in the context of ICI therapy. We should emphasize that these two terms are not mutually exclusive, and our results do not imply that NLR is only predictive for ICI and not for other types of therapy. Nevertheless, these findings expand upon prior knowledge of NLR, by indicating that this value is associated with the likelihood of clinical response to ICI therapy based on the analyses of PFS, response, and clinical benefit. These endpoints have not, to the best of our knowledge, been analyzed in large pan-cancer cohorts to date.

Nonetheless, before proceeding, there are several important caveats to these results. First, although these results demonstrate that NLR can help predict the likelihood of clinical response in cancer patients treated with ICI, these results do not rule out the possibility that NLR may also be predictive of response to other therapies. For this reason, it would be premature to prioritize patients for ICI or non-ICI therapies, based solely on NLR data. Second, we cannot conclude that the strong associations we observed in pan-cancer are equally present in all cancer types. We observed directionally similar associations between NLR and survival/response outcomes in nearly all cancer types, except for endometrial and ovarian cancer, but sample sizes for these cancer types were too small to permit definitive conclusions.

Associations between peripheral blood NLR and outcomes after ICI may be attributable to a correlation between circulating neutrophils and tumor microenvironment neutrophils^35–37^. As neutrophils can secrete immunosuppressive mediators and angiogenic factors such as reactive oxygen species, VEGF, (vascular endothelial growth factor) and MMP-9 (matrix metalloproteinase 9), neutrophil infiltration can contribute to a pro-tumor microenvironment^38,39^. Neutrophils have been associated with tumor initiation, progression, and early dissemination^40–42^. In addition, low levels of circulating lymphocytes may correlate with lower levels of tumor-infiltrating lymphocytes and a reduced anti-tumor T-cell response^6,43^. Moreover, T-cell response can be further suppressed by a higher presence of myeloid cells, including neutrophils^11,40^. All these factors create an immunosuppressive tumor microenvironment that may reduce the likelihood of response to ICI. At the same time, neutrophils in the peripheral blood have recently been shown to increase the efficiency of distant metastasis by interacting with circulating tumor cells and potentiating their metastatic phenotype through supporting cell cycle progression and accelerating metastasis seeding^44–47^.

By combining NLR with TMB—another variable with predictive value across multiple cancer types—in a simple four-category scheme based on NLR-low/high and TMB-low/high, we observed that each variable was independently associated with outcomes. This scheme defines a category of patients (NLR-low/TMB-high) with higher response rates from ICI treatment and a category of patients (NLR-high/TMB-low) with lower response rates.

Patients selected for ICI routinely undergo blood tests including absolute counts of neutrophils and lymphocytes. Therefore, NLR may be potentially widely available, minimally invasive, and measurable with a ratio that can be simply calculated from a complete blood count drawn in any setting. NLR also adds independent value to TMB, another measure observed to have pan-cancer predictive capacity.

Future considerations on the role of neutrophils in immunotherapy should be explored. Because of all the pro-tumor effects of neutrophils, as well as our results on NLR, the assessment of candidates for ICI should focus not only on the lymphoid population but also on the myeloid population. These data support investigation of new therapeutic targets that could suppress or reprogram neutrophils to potentially overcome ICI resistance^48^.

The strengths of this study include the large number of patients—compared to prior publications—along with the availability of OS, PFS, response, and clinical benefit data for every patient, and inclusion of multiple cancer types. Our results are consistent with two recent studies linking higher NLR to poorer OS in non-small cell lung cancer and other cancers^49,50^, and in addition, show an association between NLR and rates of ICI treatment response across multiple cancer types. In addition, this analysis integrates NLR with TMB across a large cohort of patients and extends prior data linking TMB to outcome by adding response data.

This study has inherent limitations including those due to its retrospective nature and the performance of the study at one center. NLR can be influenced by multiple factors, such as infections, treatment with steroids, or other stress triggers, which were not assessed in this study. Also, as NLR and TMB^51^ have distributions that vary across cancer types, it is difficult to set a universal value of NLR/TMB for stratifying patients. Both NLR and TMB are strongly associated with clinical outcome, even if the discriminatory ability of any single numerical value is more limited. These data show that with a prespecified universal cutoff, we could still stratify patients accurately, although we caution that choosing the ideal number for clinical use within each cancer type requires further validation data.

The association between high NLR and outcome was not statistically significant in all cancer types. This is likely attributable to limited statistical power in those cancers with smaller sample size. Although trends were directionally consistent among nearly all cancer types, with the exception of endometrial cancer, CIs were wide and crossed 1.0 for many subgroups. Although prior research has found that high NLR is associated with poorer prognosis in endometrial cancer^52,53^, to the best of our knowledge, no prior studies have examined this in the context of immunotherapy.

Other authors have documented changes in NLR during treatment and suggested that an increase in NLR during treatment may reflect the development of adaptive resistance to therapy^24,28,54,55^. NLR values during treatment were not available for this study; therefore, we could not examine NLR as a dynamic biomarker. While our results indicate that baseline NLR can help predict the likelihood of response to ICI, on-therapy changes in NLR may provide additional insights and should be studied further.

In summary, in this study of over 2000 patients treated with ICI, higher NLR was associated with poorer survival and lower probability of respons to immunotherapy, both at a pan-cancer level and within several cancer types. NLR is a minimally invasive, affordable, and readily available biomarker. As a sole marker, NLR values were associated with the probability of response to ICI. If TMB is also available, the combination of both factors provides additional predictive value in patients treated with ICI. Further study will be needed to confirm that this is a broadly applicable biomarker in cancer patients. Future treatment strategies targeting the myeloid population may help overcome ICI resistance.

## Methods

### Patient selection

The main study questions (whether NLR and TMB are associated with response, clinical benefit, PFS, and OS) were specified before data collection began. Patients initially selected for the study were all those with solid tumors diagnosed from 2015 through 2018, who received at least one dose of ICI at our center (*N* = 2827). All tumors, along with DNA from peripheral blood, were genomically profiled using the MSK-IMPACT next-generation sequencing platform^56^. All patients provided written informed consent for tumor sequencing. This study was approved by the Memorial Sloan Kettering Cancer Center institutional review board.

We excluded patients with history of more than 1 cancer, those without a complete blood count within 30 days prior to the first dose of ICI, those enrolled in blinded trials, and cancer types with fewer than 25 cases. The clinical records of the remaining 1854 patients were manually reviewed to assess response to therapy, PFS, and OS. The process was blinded to patients’ NLR and TMB. We excluded patients who received ICI in a neoadjuvant or adjuvant setting, and patients with unevaluable response (lost to follow-up without imaging after ICI start). The final cohort consisted of 1714 patients with 16 cancer types (Supplementary Fig. 1 and Supplementary Table 1).

### Clinical and genomic data

NLR was calculated as the absolute count of neutrophils neutrophils (per nL) divided by the absolute count of lymphocytes (per nL). NLR values were gathered from the closest blood test prior to the first ICI infusion. TMB, derived from DNA in tumor tissue, was defined as the total number of somatic non-synonymous tumor mutations normalized to the exonic coverage of the respective MSK-IMPACT panel in megabases (mutations/megabase)^2^. Samples in this study were profiled with the MSK-IMPACT 410 gene (*n* = 482) or 468 gene (*n* = 1232) panels (panel versions and genes are listed in Supplementary Data 1). Out of the 1714 patients, 1623 (95%) had MSK-IMPACT on a sample collected prior to ICI start and 91 (5%) on a sample collected after ICI start. For patients for whom MSK-IMPACT was performed on a sample collected prior to ICI start, the median time between the sample collection and ICI start was 6 months (IQR 1–12). Clinical covariates analyzed were age at ICI first infusion, sex, cancer type, ICI drug class, stage at ICI first infusion, year of treatment start, performance status, and ICI line of treatment. Cancers were staged according to American Joint Committee on Cancer 8th Edition^57^. Clinical and genomic data are reported in this study following REMARK (Reporting recommendations for tumor marker prognostic studies) guidelines and are available in Supplementary Data 1.

### Validation cohort

After completion of all analyses and initial peer review of the manuscript, we obtained data for an independent cohort of 323 additional patients treated at our center, for validation of the NLR thresholds analyzed in the primary cohort. This cohort used identical inclusion and exclusion criteria as the primary cohort, but extended the years of eligibility to patients treated between 2014 and 2019, including patients with a history of only one solid tumor, with a complete blood count within 30 days prior to the first dose of ICI, and who underwent MSK-IMPACT sequencing. The characteristics of the validation cohort are shown in Supplementary Table 2.

### Study outcomes

The primary study outcomes were OS, PFS, response to ICI, and clinical benefit. We categorized response and clinical benefit based on RECIST v1.1 criteria^58^. If formal RECIST reads were not available, we manually reviewed physician notes and imaging studies to categorize overall best response for each patient using the same criteria based on change in the sum of diameters of target lesions. Response was defined as complete or partial response. Clinical benefit was defined as complete response, partial response, or stable disease for ≥6 months. PFS was calculated from ICI first infusion to disease progression or death of any cause; patients without progression were censored at last attended appointment at MSK with any clinician. OS was calculated from ICI first infusion to death of any cause; patients alive at time of review were censored at last contact. For patients who received multiple lines of ICI, the first line was used for analysis.

### Statistical analyses

NLR distribution across cancer types was analyzed using density plots. To account for differences in NLR and TMB distributions across cancer types, we calculated NLR and TMB percentiles within each cancer type. Top 20th percentile was selected as the definition of NLR-high or TMB-high^2^. Clinical benefit trends based on NLR and TMB were plotted using LOWESS models. To test for correlation between NLR and TMB, we calculated Spearman’s correlation coefficient. To analyze the degree of discrimination of NLR and TMB, we performed ROC and AUC analysis. To analyze the combined effect of NLR and TMB, we assigned patients into four equal-sized categories of NLR low/high and TMB low/high, using median within cancer type. Analyses were first performed pan-cancer (i.e., across the entire study population) and then stratified by cancer type.

Differences in response and clinical benefit between groups were compared using Pearson’s *χ*^2^-test or Fisher’s exact test. Survival was calculated according to the Kaplan–Meier method and compared between groups using the log-rank test. HRs were calculated according to Cox’s proportional hazard regression model. ORs were calculated according to logistic regression. All variables significant in univariate analysis were included in multivariable analyses as covariates. A *P*-value of less than 0.05 was considered statistically significant, and all hypothesis tests were two-sided. Clinical and genomic data for all patients were reviewed between June and September of 2019. All statistical analyses were conducted using Stata (Stata Statistical Software: Release 16; StataCorp LLC).

### Reporting summary

Further information on research design is available in the Nature Research Reporting Summary linked to this article.

## Supplementary information

Supplementary Information

Description of Additional Supplementary Files

Supplementary Data 1

Reporting Summary

## Data Availability

All data needed to replicate the analyses in this study, including de-identified clinical and outcomes data, NLR and TMB values, and details of the genes on the IMPACT panel, are provided in Supplementary Data 1. Original sequencing reads cannot be publicly deposited due to privacy restrictions, as sequencing was performed as part of clinical care.
