# Supplementary material for: Pretreatment neutrophil-to-lymphocyte ratio and mutational burden as biomarkers of tumor response to immune checkpoint inhibitors
Source: Nat Commun. 2021 Feb 1;12:729. doi: 10.1038/s41467-021-20935-9 (PMC7851155; doi:10.1038/s41467-021-20935-9)
Supplement: Supplementary file 1 — Supplementary Information [file 41467_2021_20935_MOESM1_ESM.pdf]

## Supplementary Figure 1. Flow diagram

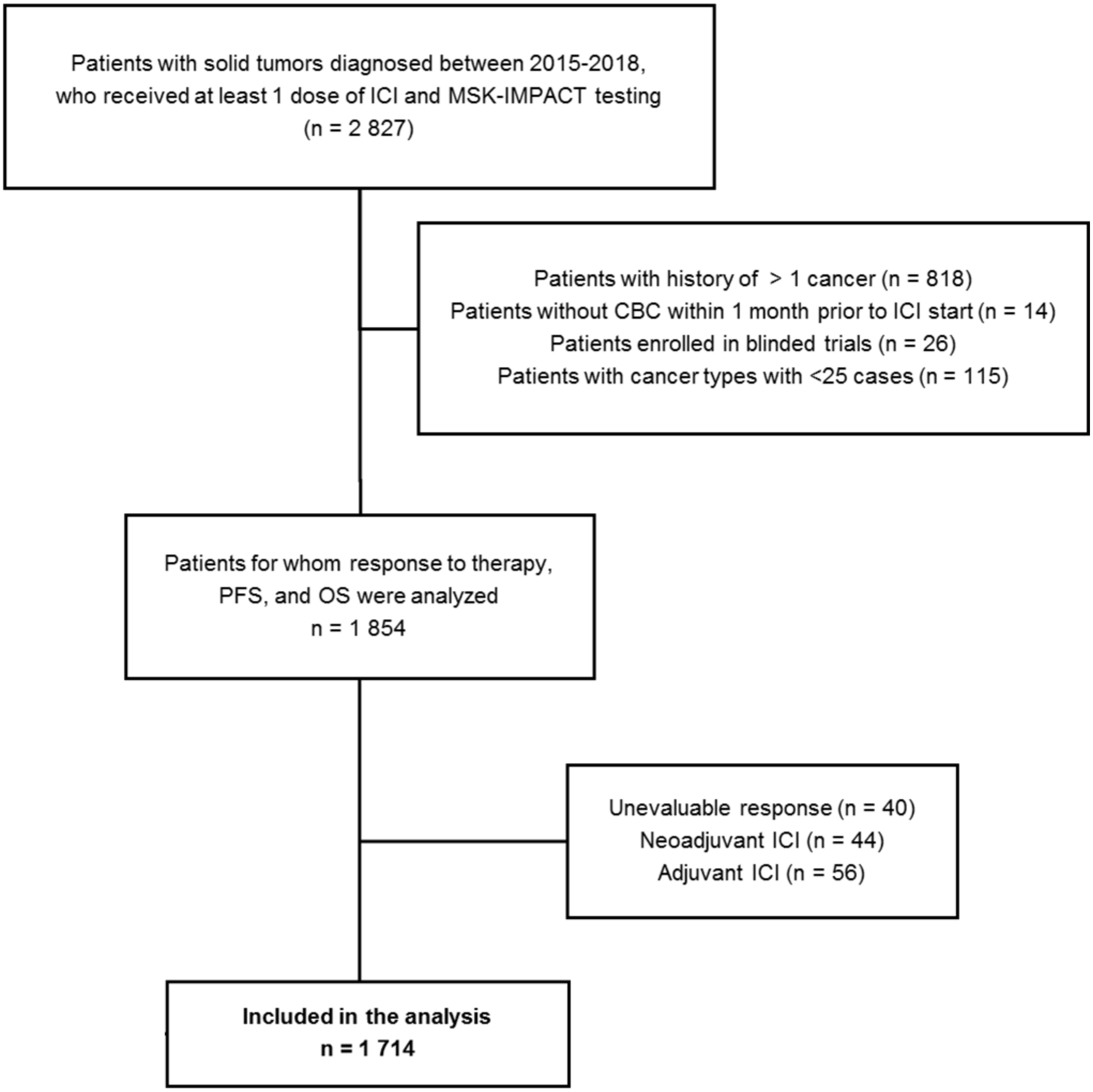


Abbreviations: ICI, Immune checkpoint inhibitors; CBC, complete blood count; PFS, progression-free survival; OS, overall survival

**Supplementary Figure 2. Neutrophil-to-lymphocyte ratio (NLR) distribution according to cancer type**

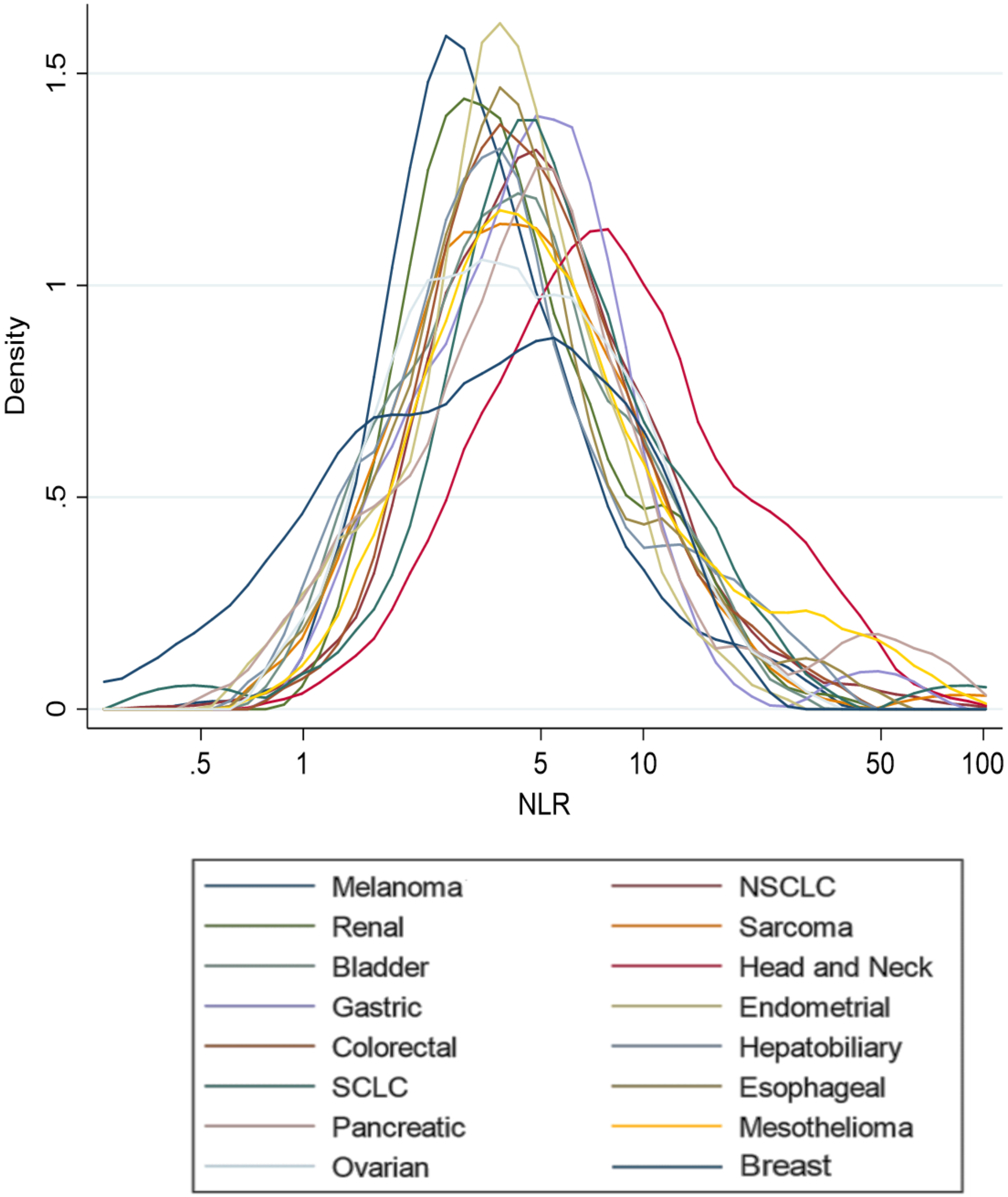


Kernel density plots of neutrophil-to-lymphocyte ratio (log scale) across cancer types.

Abbreviations: NSCLC, Non-small cell lung cancer; SCLC, small cell lung cancer

**Supplementary Figure 3. Pan-cancer overall survival according to neutrophil-to-lymphocyte ratio (NLR) percentile within cancer type**

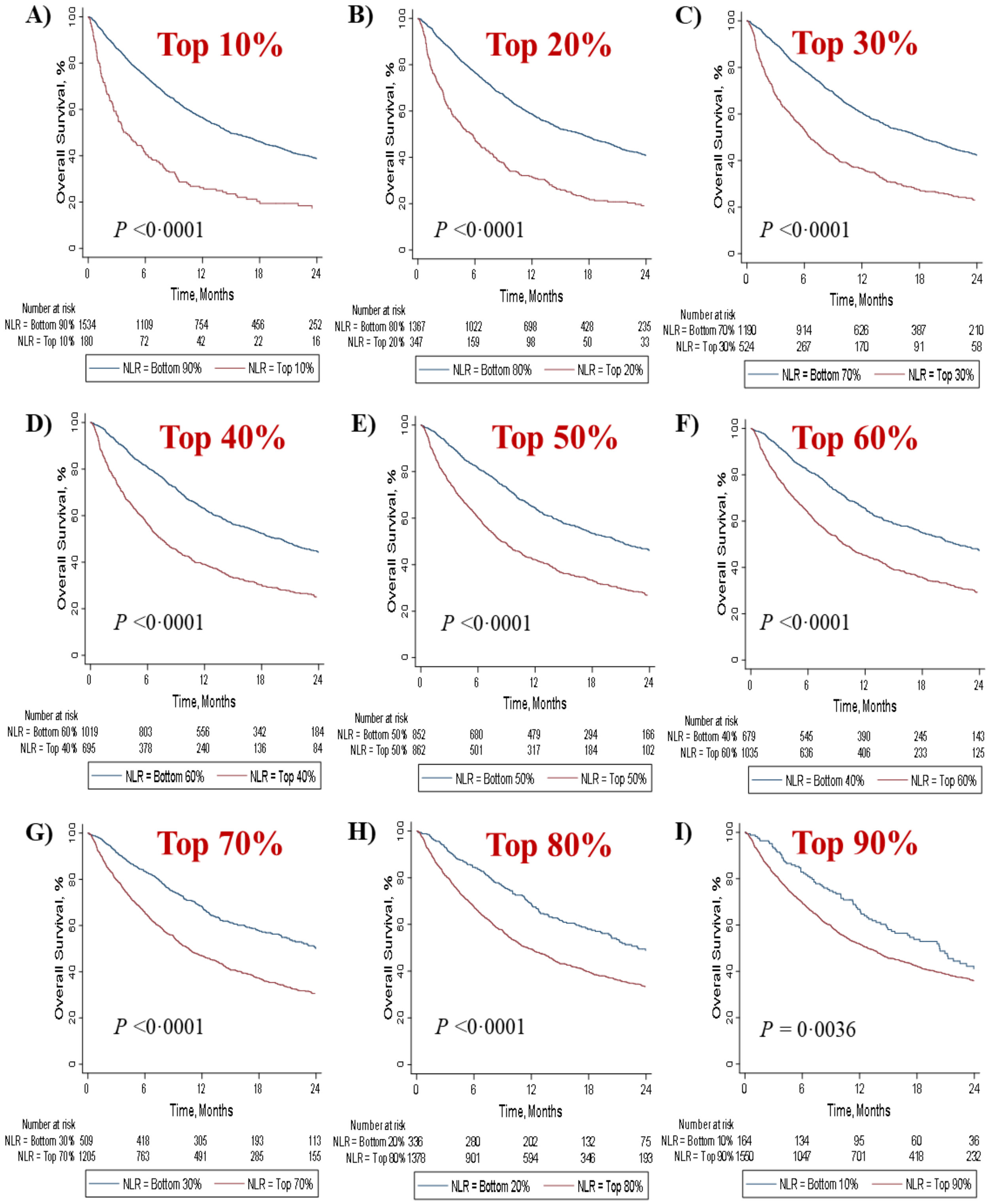


Pan-cancer overall survival comparing NLR high vs low groups, using as cutoff A) top 10th, B) top 20th, C) top 30th, D) top 40th, E) top 50th, F) top 60th, G) top 70th, H) top 80th and I) top 90th percentile within cancer type. P values are according to log-rank test.

**Supplementary Figure 4. Pan-cancer progression-free survival according to neutrophil-to-lymphocyte ratio (NLR) percentile within cancer type**

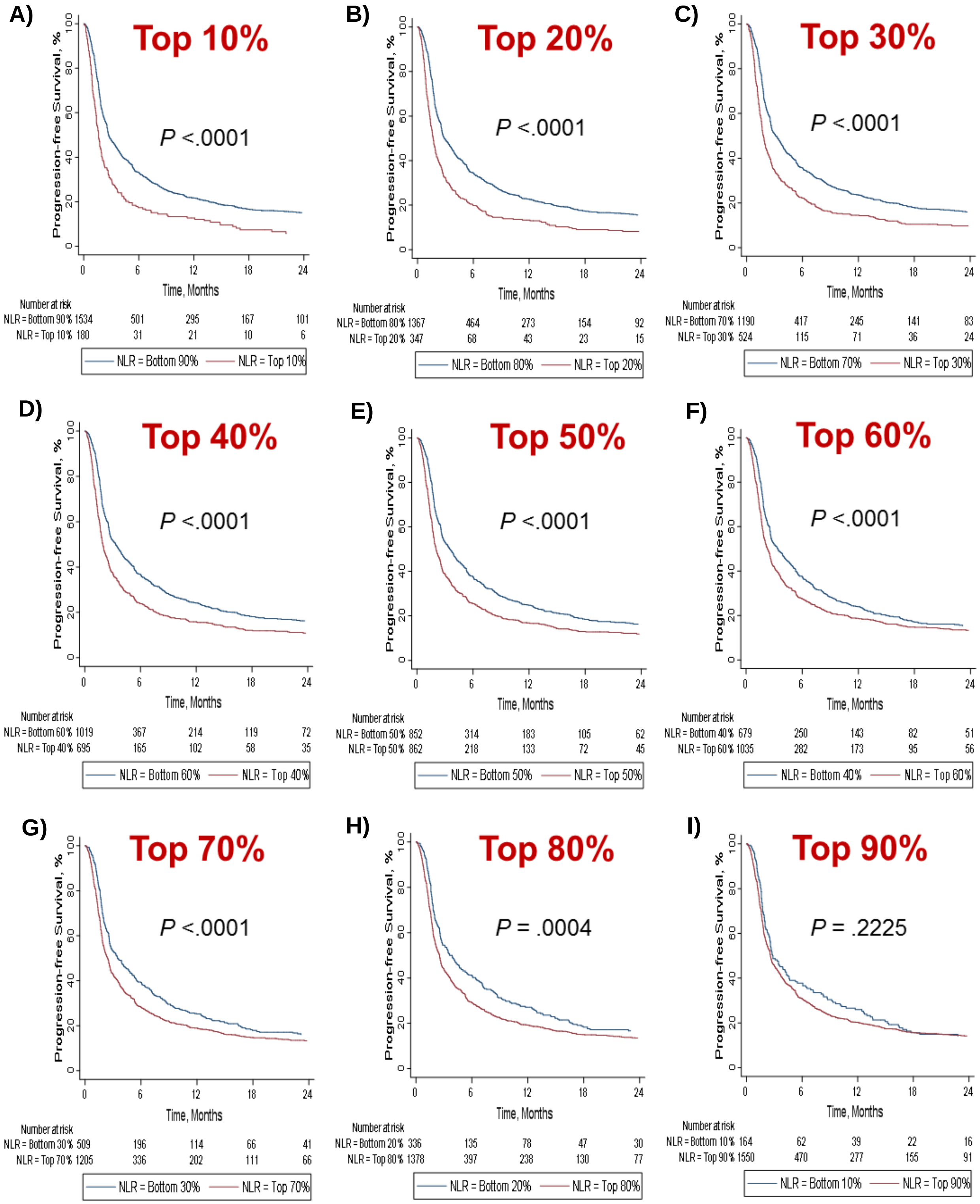


Pan-cancer progression-free survival comparing NLR high vs low groups, using as cutoff A) top 10th, B) top 20th, C) top 30th, D) top 40th, E) top 50th, F) top 60th, G) top 70th, H) top 80th and I) top 90th percentile within cancer type. P values are according to log-rank test.

**Supplementary Figure 5. Pan-cancer clinical benefit according to neutrophil-to-lymphocyte ratio (NLR) percentile within cancer type**

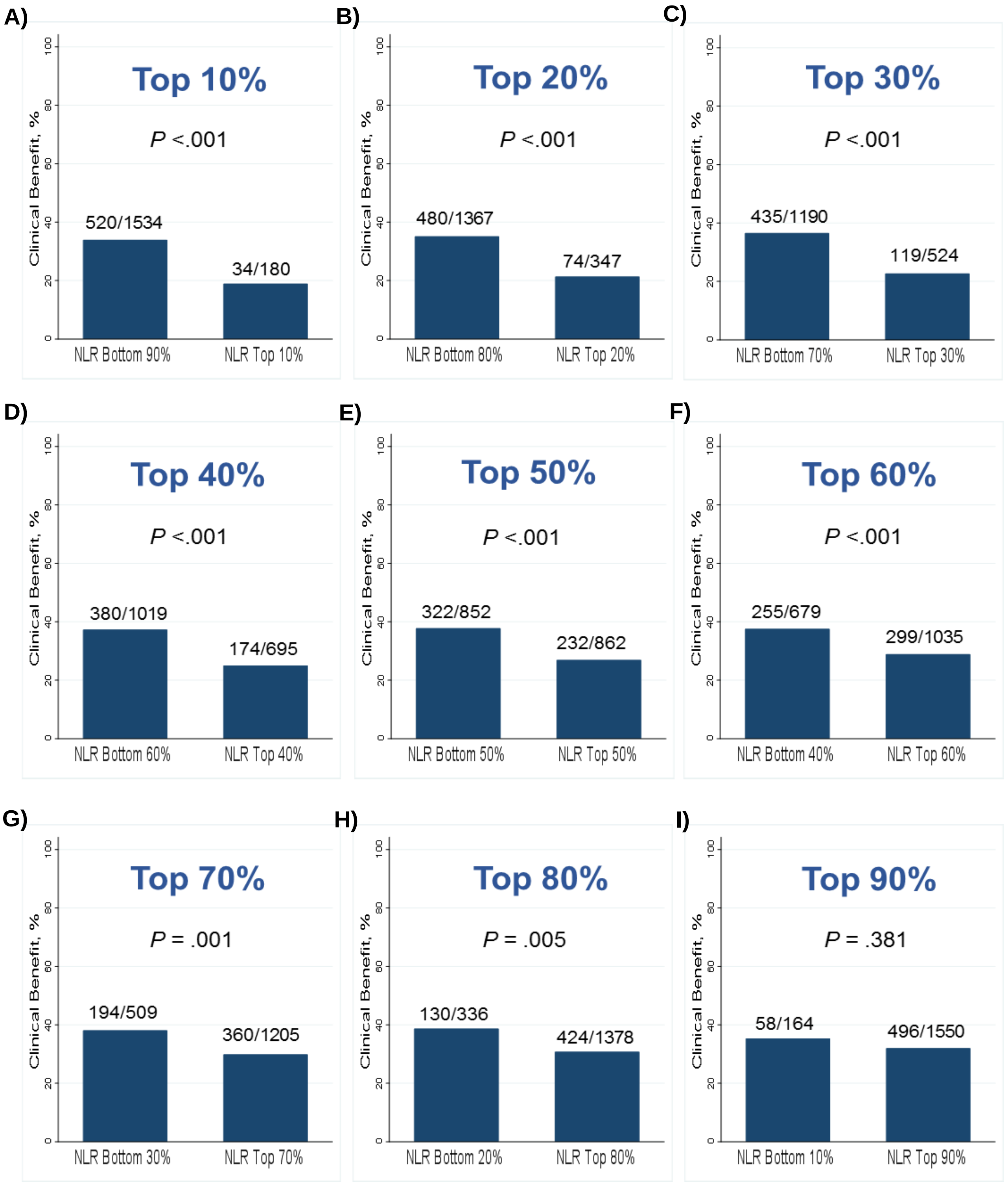


Pan-cancer clinical benefit comparing NLR high vs low groups, using as cutoff A) top 10th, B) top 20th, C) top 30th, D) top 40th, E) top 50th, F) top 60th, G) top 70th, H) top 80th and I) top 90th percentile within cancer type. P values are according to Pearson´s chi-square test.

**Supplementary Figure 6. Pan-cancer response according to neutrophil-to-lymphocyte ratio (NLR) percentile within cancer type**

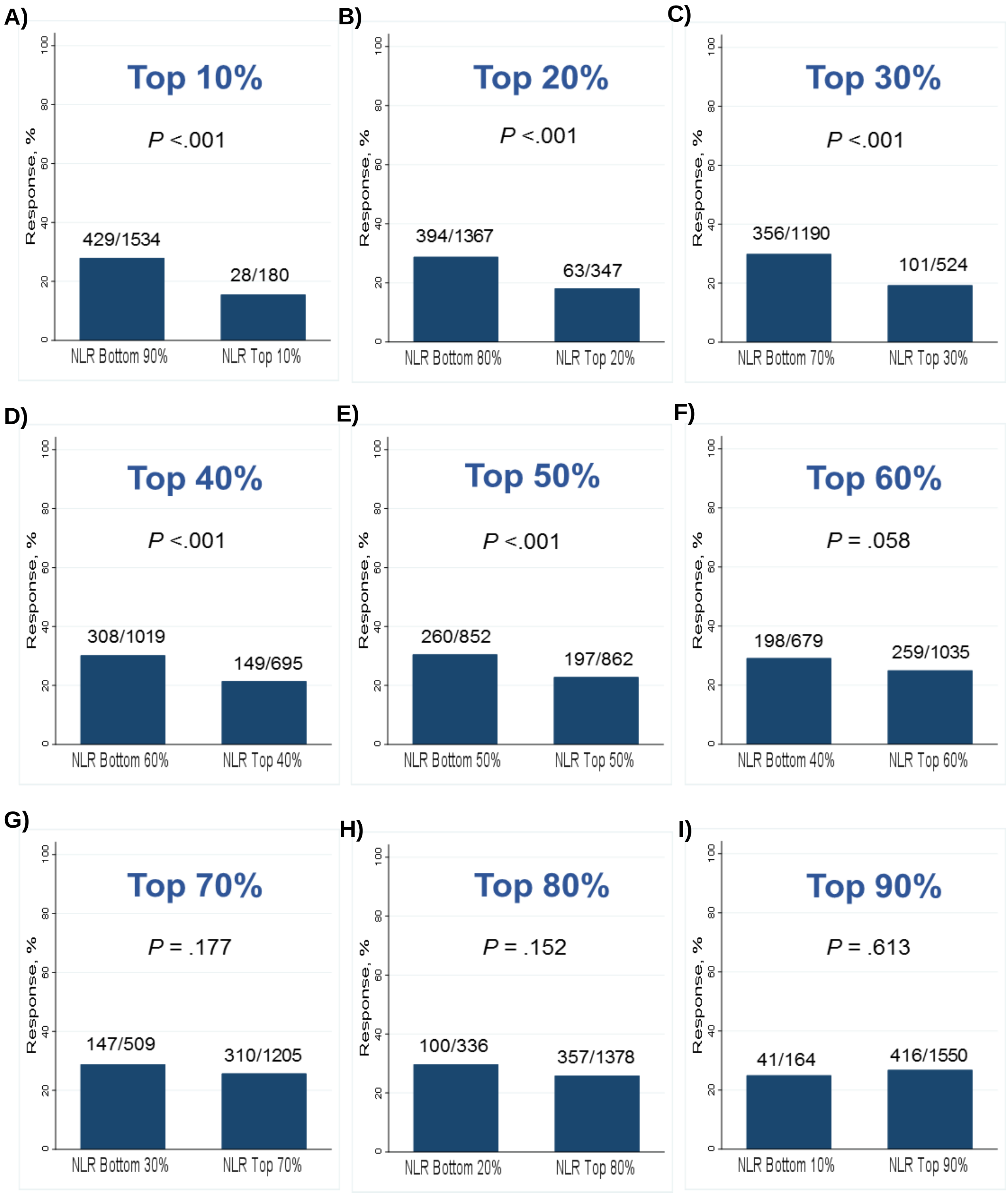


Pan-cancer response comparing NLR high vs low groups, using as cutoff A) top 10th, B) top 20th, C) top 30th, D) top 40th, E) top 50th, F) top 60th, G) top 70th, H) top 80th and I) top 90th percentile within cancer type. P values are according to Pearson´s chi-square test.

**Supplementary Figure 7. Outcomes based on neutrophil-to-lymphocyte ratio (NLR) using top 20th percentile within cancer type as cutoff and stratified by cancer type**

A)

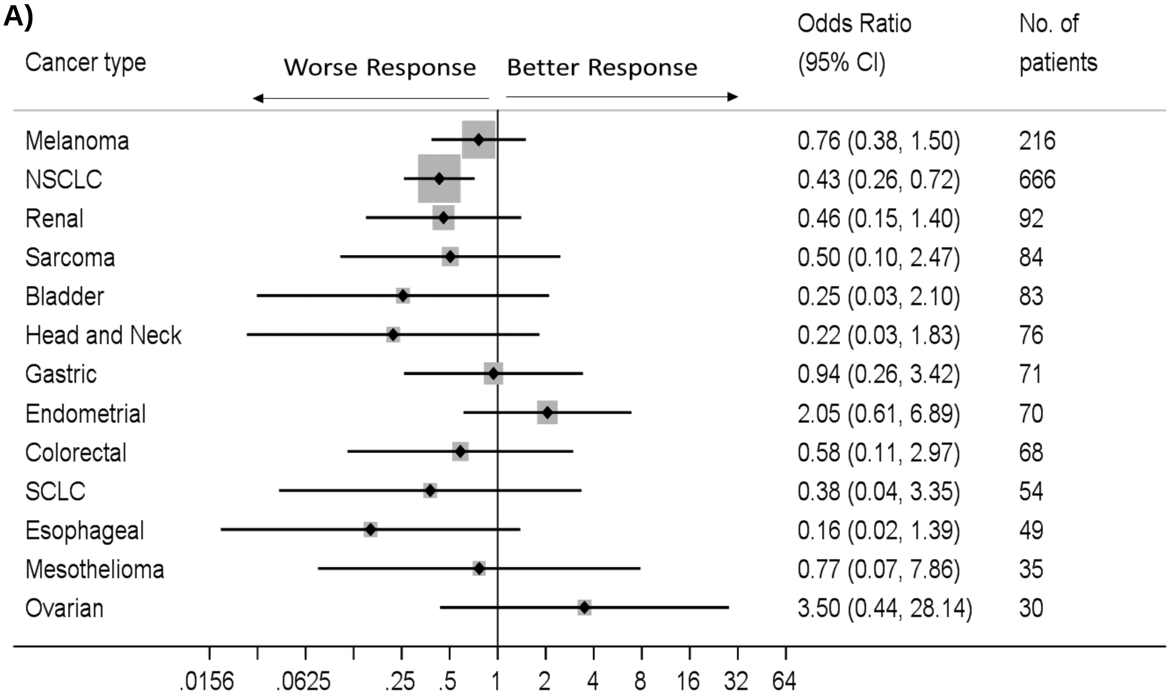


B)

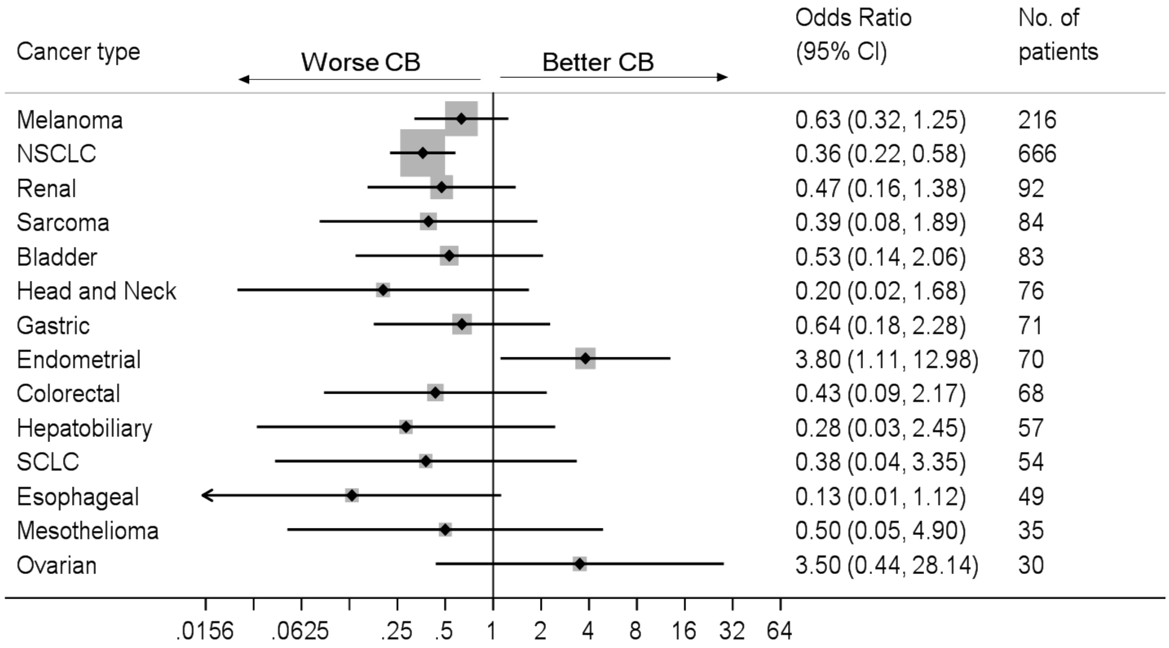


Forest plots showing odds ratios with 95% CIs for A) response and B) clinical benefit across cancer types comparing top 20th NLR percentile within cancer type to bottom 80th. Odds ratios were calculated with logistic regression. Logistic regression models for response are unstable for hepatobiliary, pancreatic and breast and are not included in figure A. Logistic regression models for clinical benefit are unstable for pancreatic and breast and are not included in figure B.

Abbreviations: CI, Confidence interval; NSCLC, non-small cell lung cancer; SCLC, small cell lung cancer; CB, clinical benefit

**Supplementary Figure 8. Pan-cancer outcomes based on neutrophil-to-lymphocyte ratio (NLR) using a universal cutoff (NLR ≥ 5)**

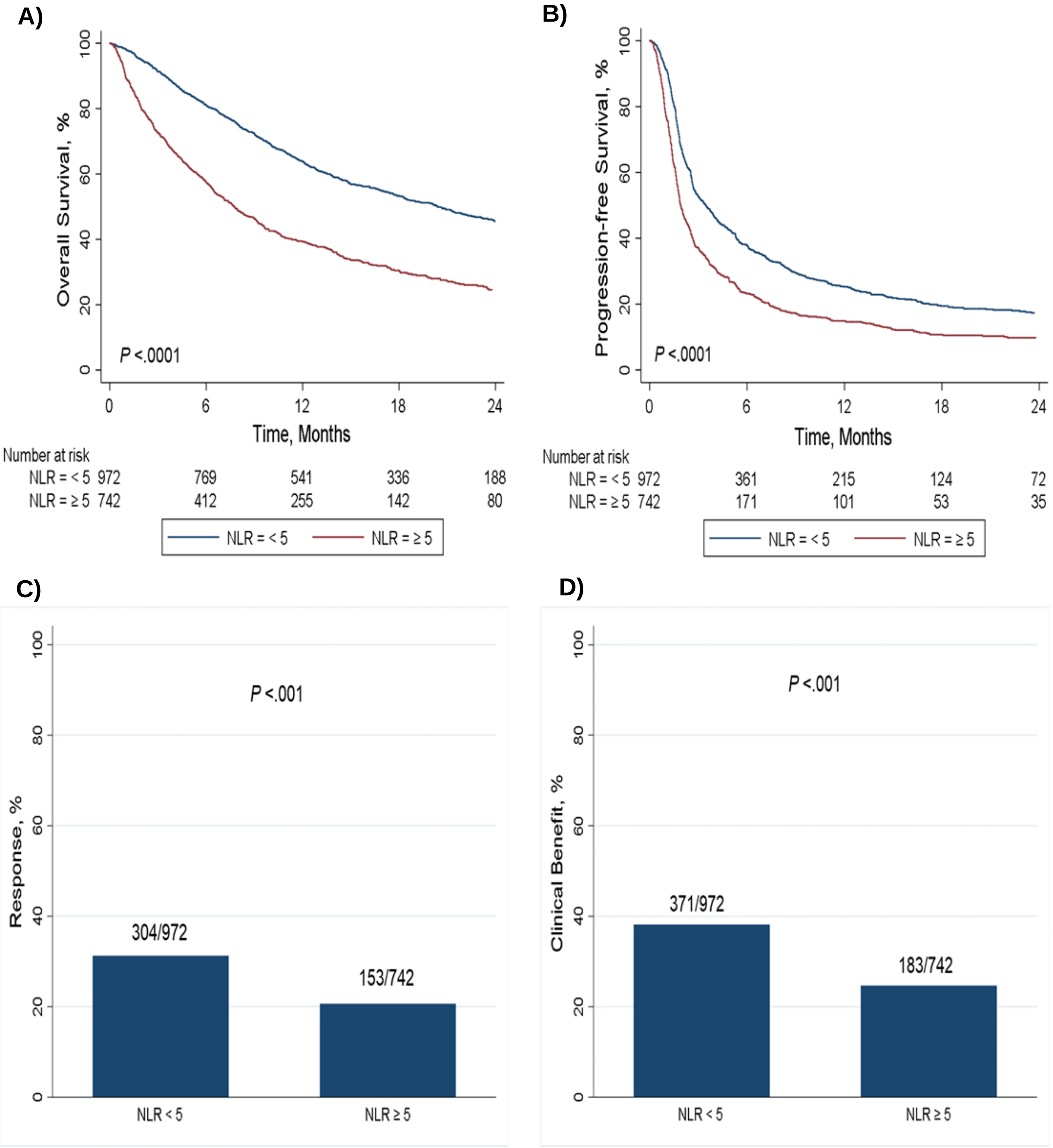


A) Overall survival, B) progression-free survival, C) response, and D) clinical benefit at a pan-cancer level based on high vs low NLR. The cutoff was ≥ 5. *P* values are according to log-rank test for A) and B) and Pearson´s chi-square test for C) and D).

**Supplementary Figure 9. Outcomes based on neutrophil-to-lymphocyte ratio (NLR) using a universal cutoff (NLR ≥ 5) and stratified by cancer type**

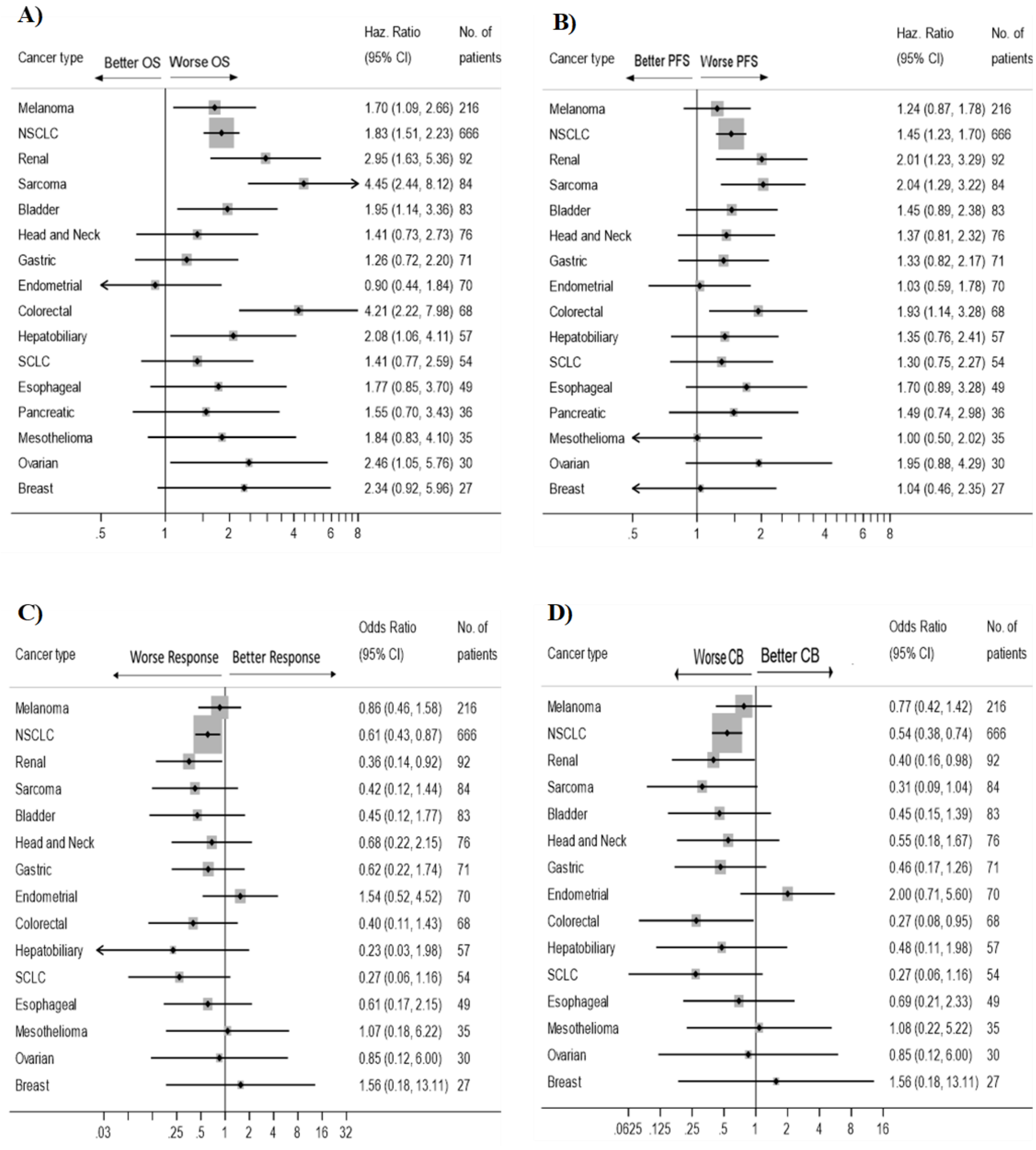


Forest plots showing hazard ratios (HRs) with 95% CIs for A) overall survival and B) progression-free survival, and odds ratios (ORs) with 95% CIs for C) response and D) clinical benefit across cancer types using universal cutoff of 5. HRs were calculated with Cox proportional hazard regression. ORs were calculated with logistic regression. Logistic regression models for response and clinical benefit are unstable for pancreatic cancer and are not included in figure C and D.

Abbreviations: CI, Confidence interval; NSCLC, non-small cell lung cancer; SCLC, small cell lung cancer

**Supplementary Figure 10. Pan-cancer outcomes based on neutrophil-to-lymphocyte ratio (NLR) in the validation cohort (n = 323)**

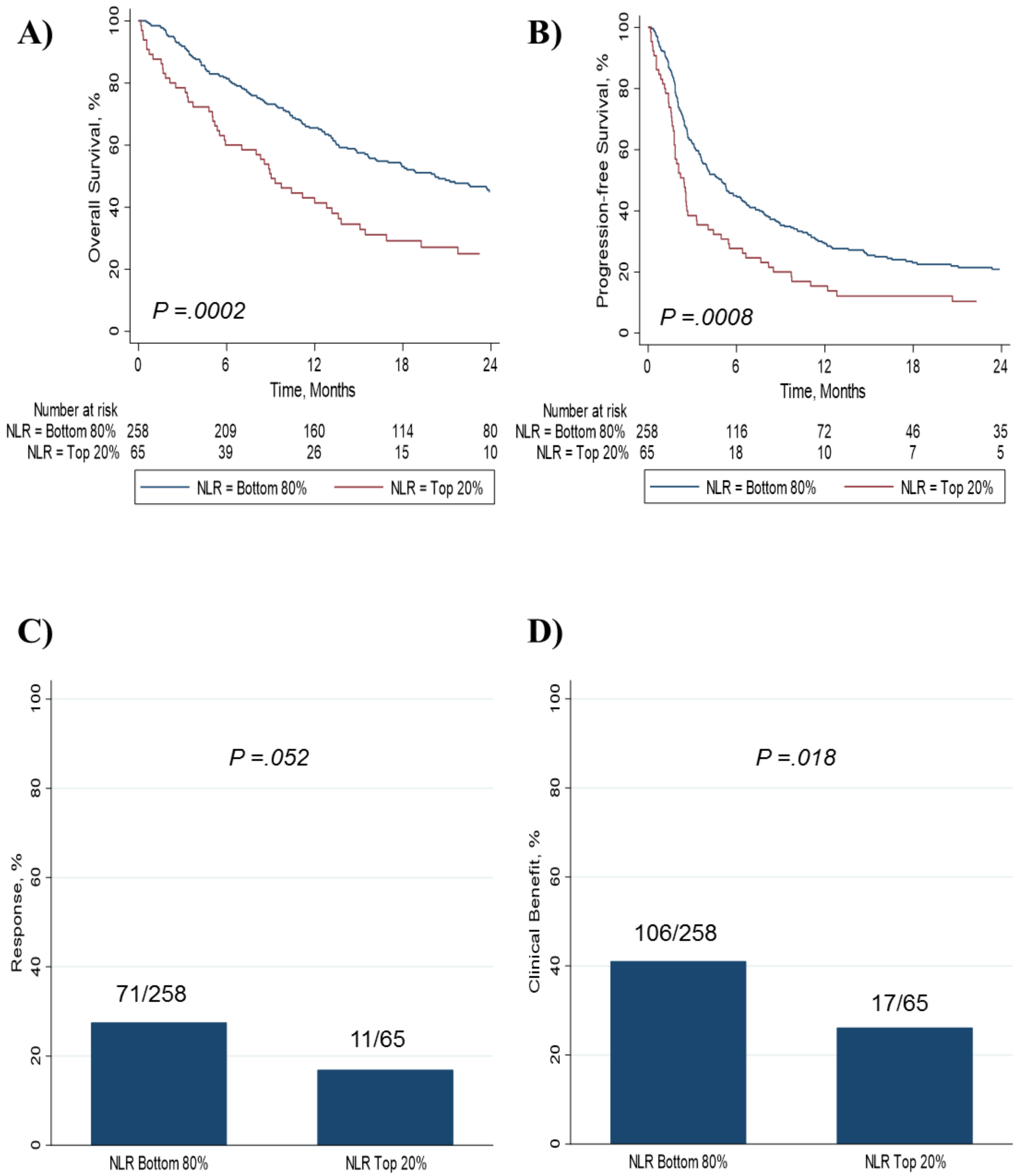


A) Overall survival, B) progression-free survival, C) response, and D) clinical benefit at a pan-cancer level based on high vs low NLR in the validation cohort (n = 323). The cutoff was the top 20th percentile within each cancer type. P values are according to log-rank test for A) and B) and Fisher's exact test for C) and D).

**Supplementary Figure 11. Clinical benefit trends based on neutrophil-to-lymphocyte ratio (NLR) and tumor mutational burden (TMB)**

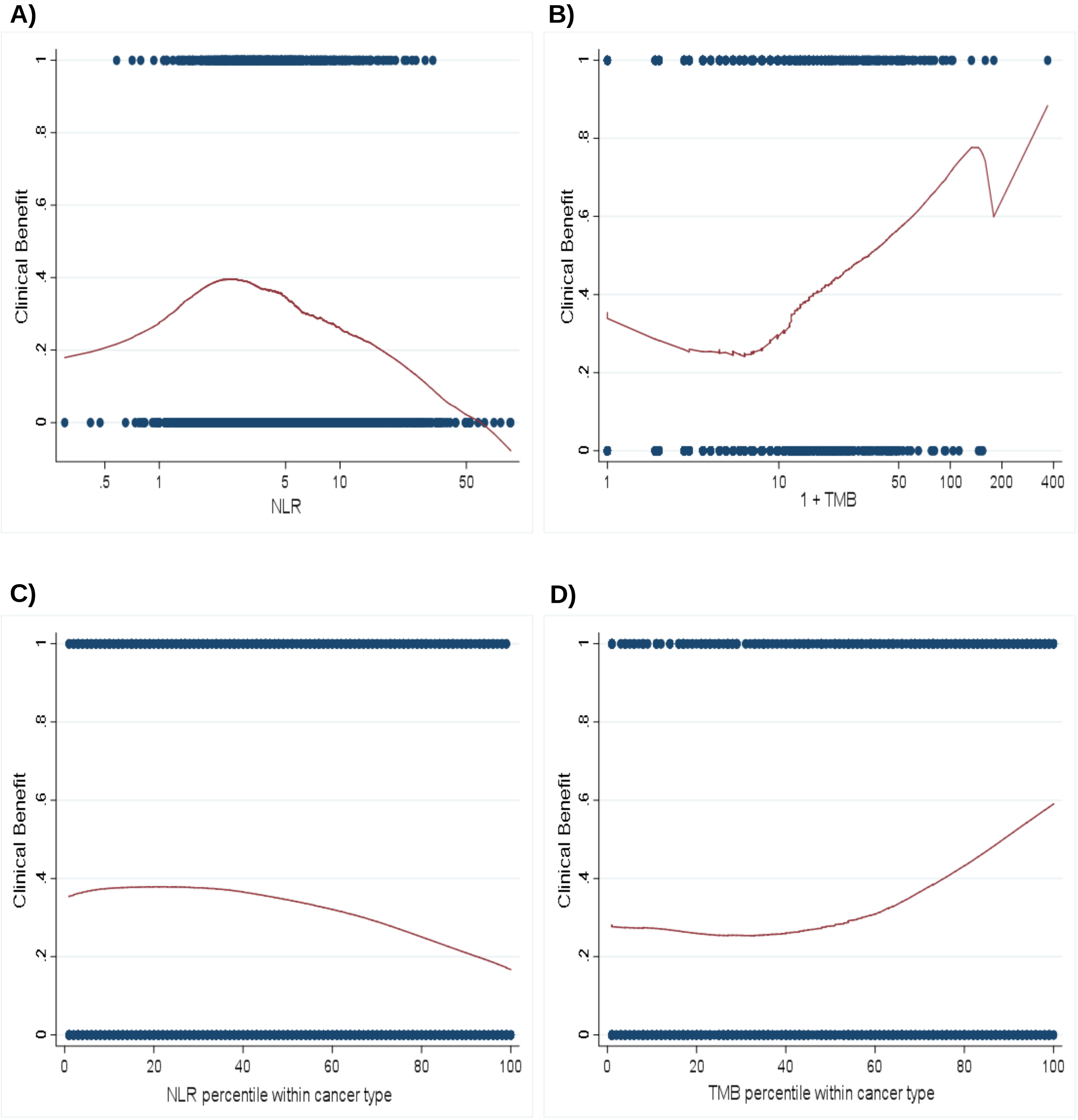


LOWESS plots of clinical benefit based on A) NLR (log scale), B) TMB (log scale), C) NLR percentile within cancer type, and D) TMB percentile within cancer type.

**Supplementary Figure 12. Correlation between neutrophil-to-lymphocyte ratio (NLR) and tumor mutational burden (TMB)**

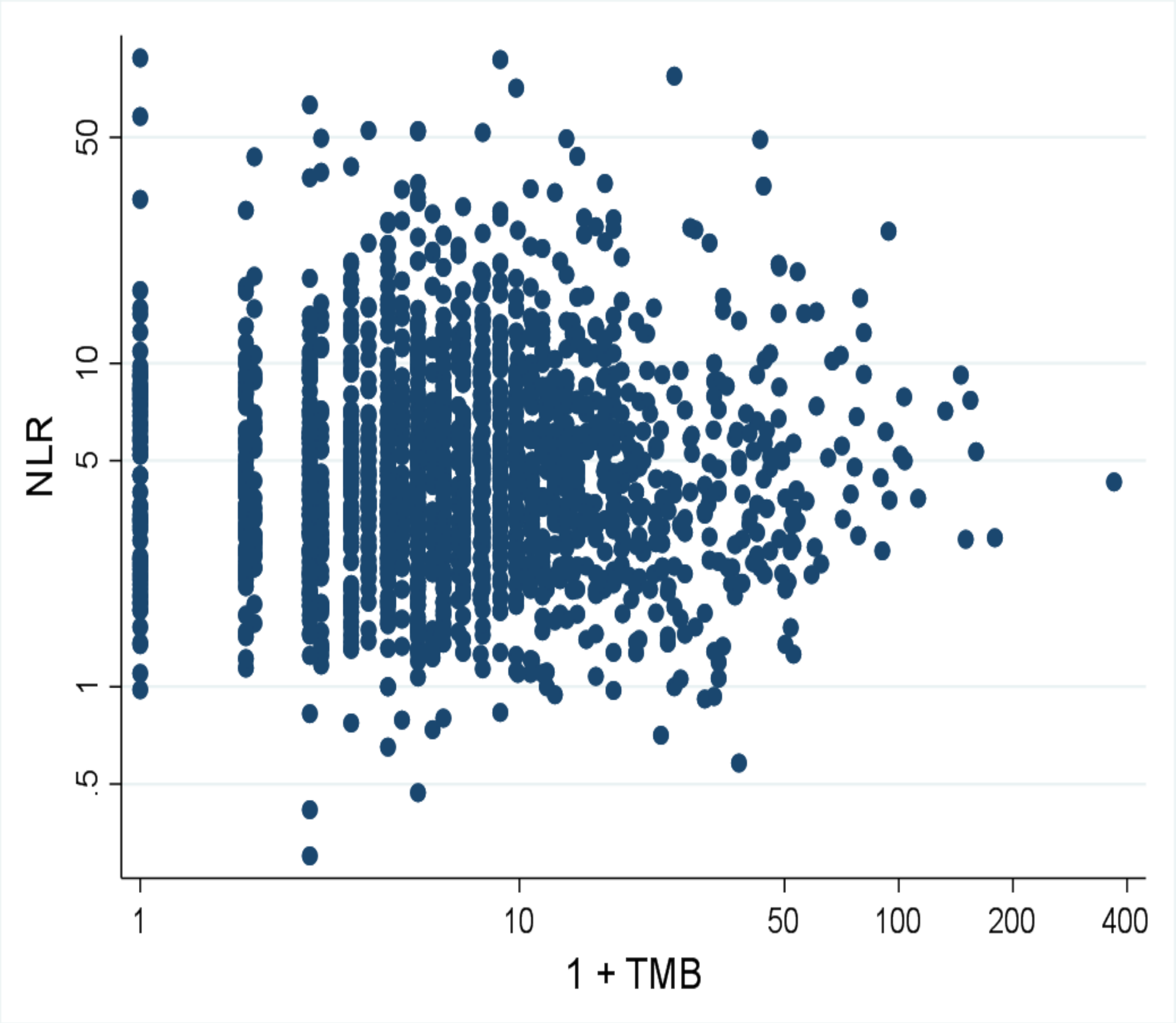


Scatter plot analyzing the correlation between NLR and TMB, both in log scale. Spearman correlation coefficient = 0.048; P = 0.05.

**Supplementary Figure 13. Receiver operating characteristic (ROC) curves for neutrophil-to-lymphocyte ratio (NLR) and tumor mutational burden (TMB) with clinical benefit as endpoint**

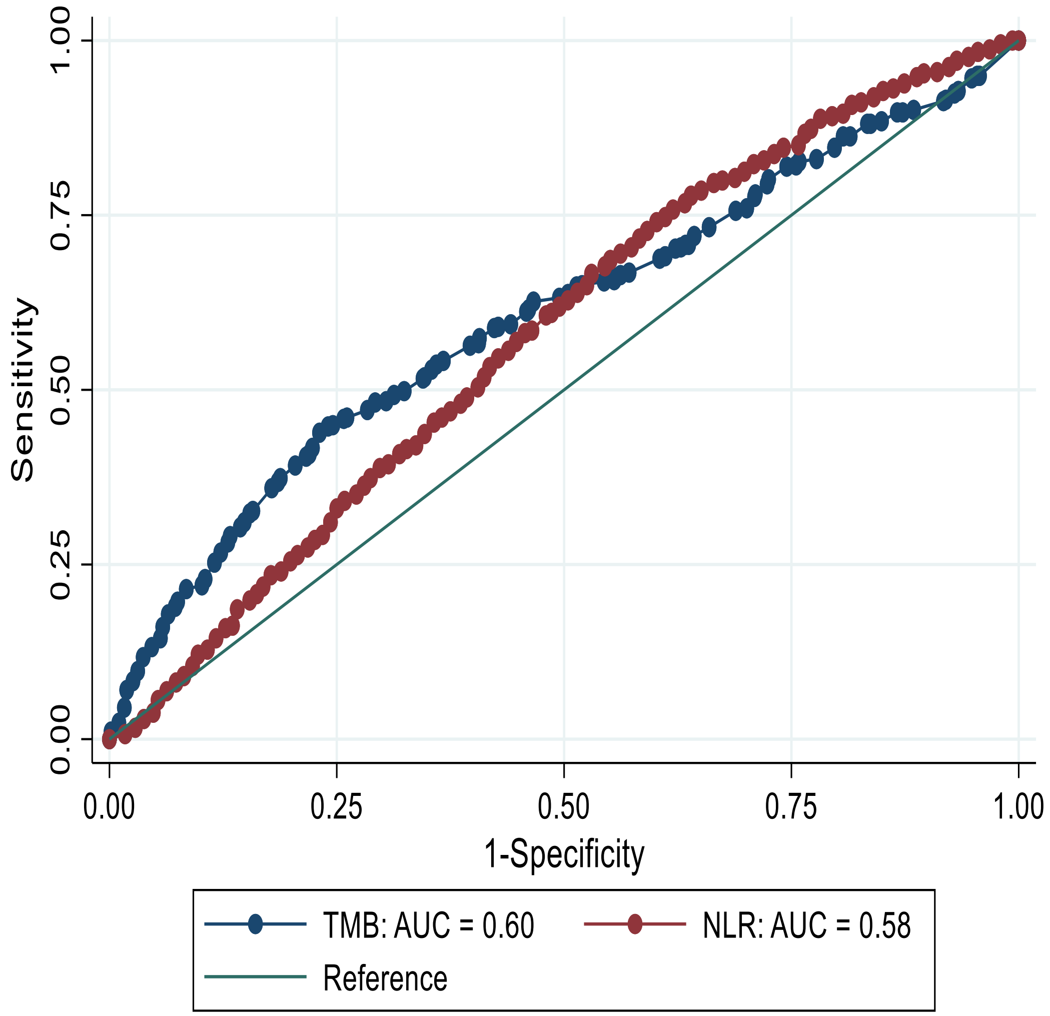


NLR and TMB expressed as percentile within cancer type.

Abbreviations: AUC, Area under the curve

## Supplementary Figure 14. Overall survival according to combined neutrophil-to-lymphocyte ratio (NLR) and tumor mutational burden (TMB) categorization for the main cancer types

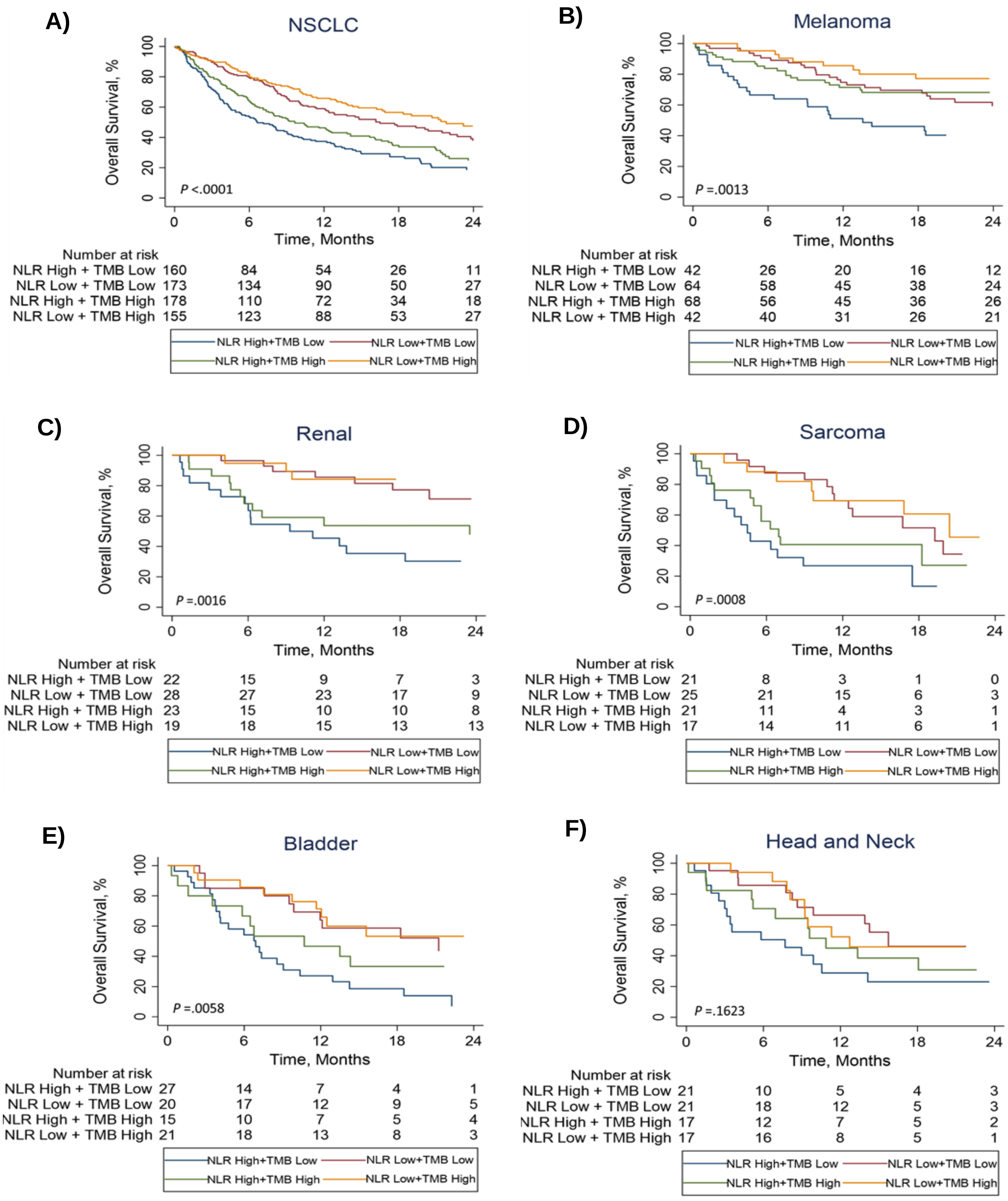


Overall survival based on a combined NLR and TMB variable stratified by cancer type for the main cancer types; A) non-small cell lung cancer (NSCLC), B) melanoma, C) renal, D) sarcoma, E) bladder, F) head and neck. We assigned patients into 4 categories based on NLR low/high and TMB low/high using median within cancer type as cutoff for both variables. P values are according to log-rank test.

**Supplementary Figure 15. Progression-free survival according to combined neutrophil-to-lymphocyte ratio (NLR) and tumor mutational burden (TMB) categorization for the main cancer types**

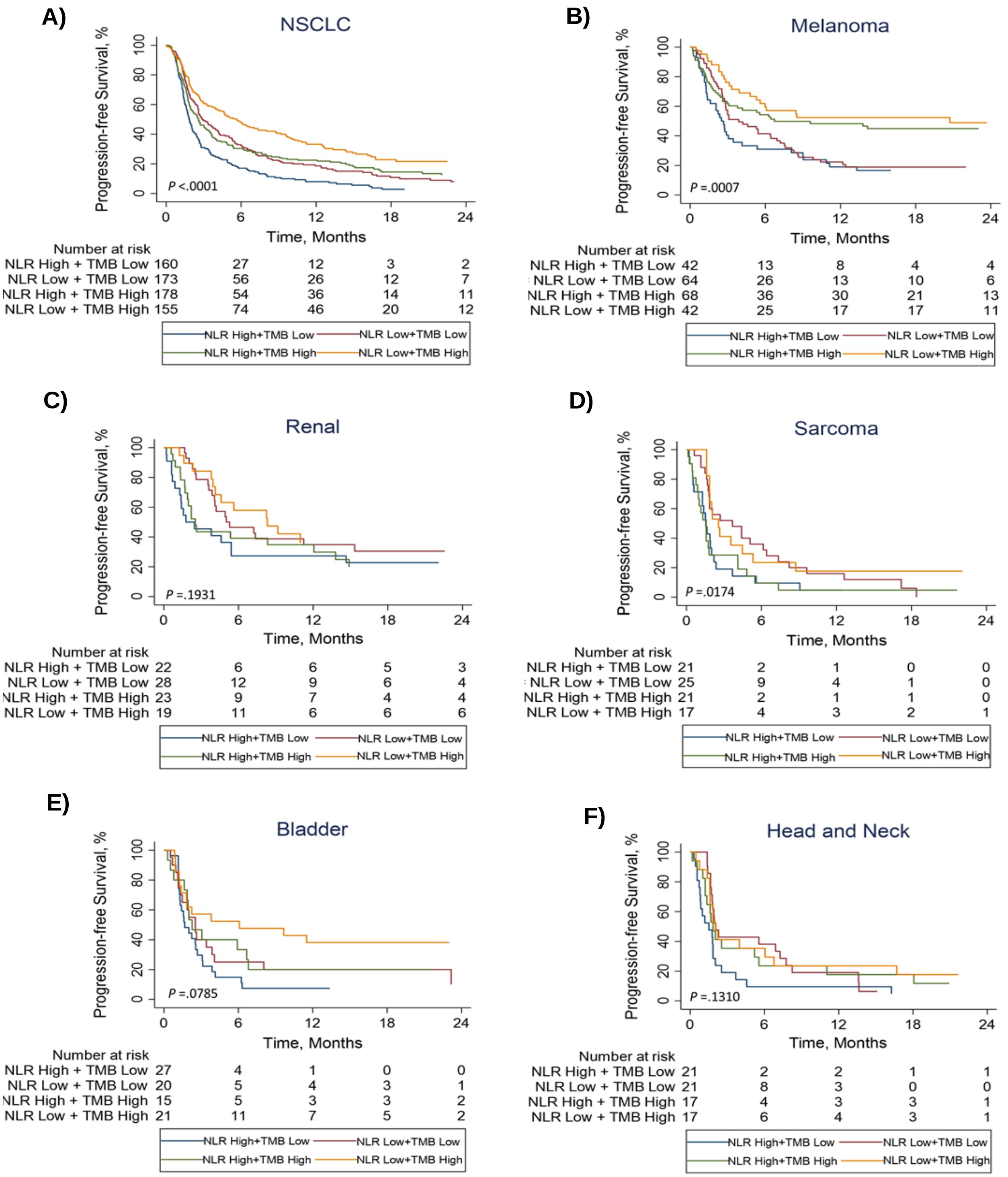


Progression-free survival based on a combined NLR and TMB variable stratified by cancer type for the main cancer types; A) non-small cell lung cancer (NSCLC), B) melanoma, C) renal, D) sarcoma, E) bladder, F) head and neck. We assigned patients into 4 categories based on NLR low/high and TMB low/high using median within cancer type as cutoff for both variables. P values are according to log-rank test.

**Supplementary Figure 16. Response according to combined neutrophil-to-lymphocyte ratio (NLR) and tumor mutational burden (TMB) categorization for the main cancer types**

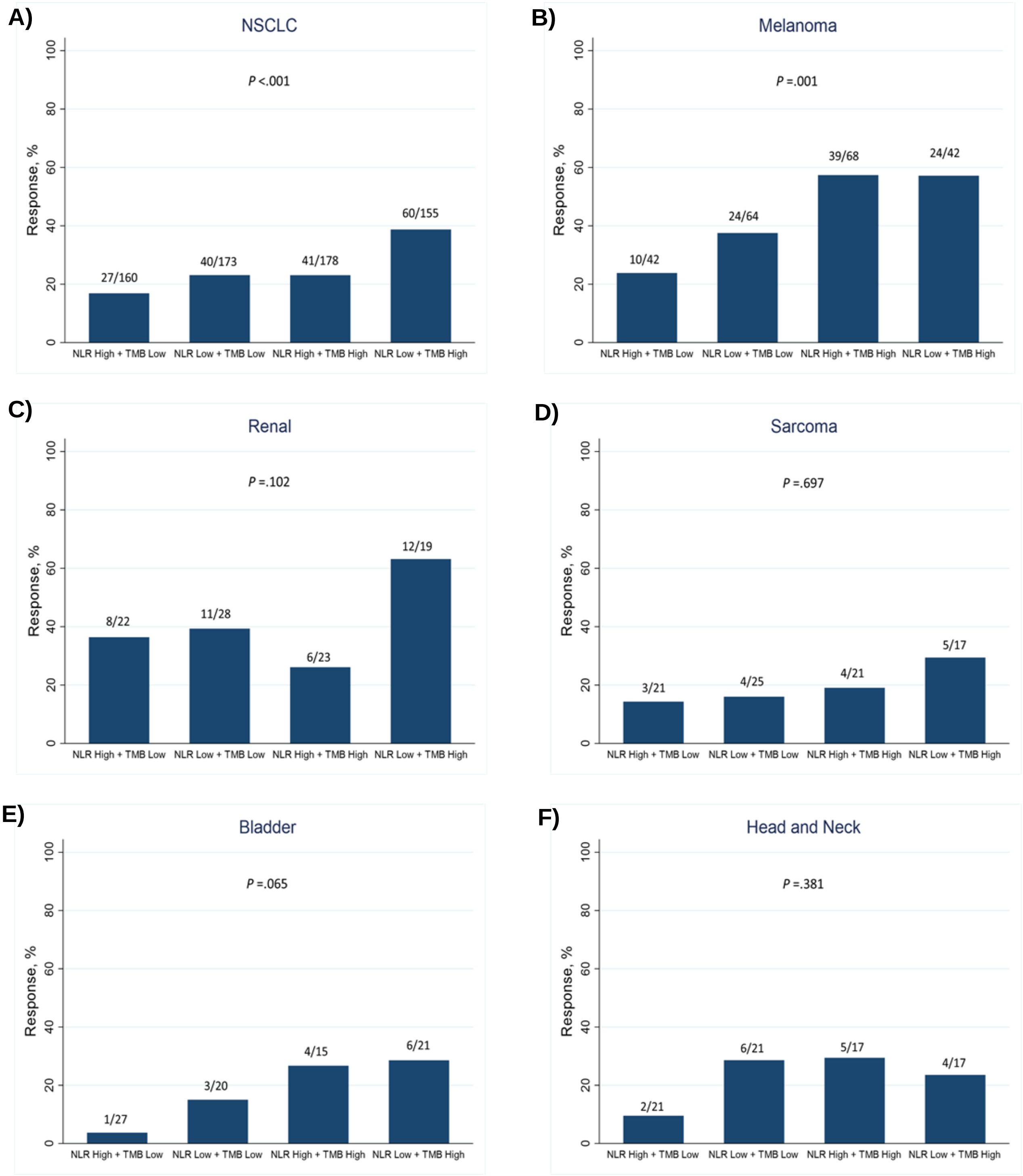


Response based on a combined NLR and TMB variable stratified by cancer type for the main cancer types; A) non-small cell lung cancer (NSCLC), B) melanoma, C) renal, D) sarcoma, E) bladder, F) head and neck. We assigned patients into 4 categories based on NLR low/high and TMB low/high using median within cancer type as cutoff for both variables; P values according to Pearson´s chi-square test or Fisher´s exact test when appropriate.

**Supplementary Figure 17. Clinical benefit according to combined neutrophil-to-lymphocyte ratio (NLR) and tumor mutational burden (TMB) categorization for the main cancer types**

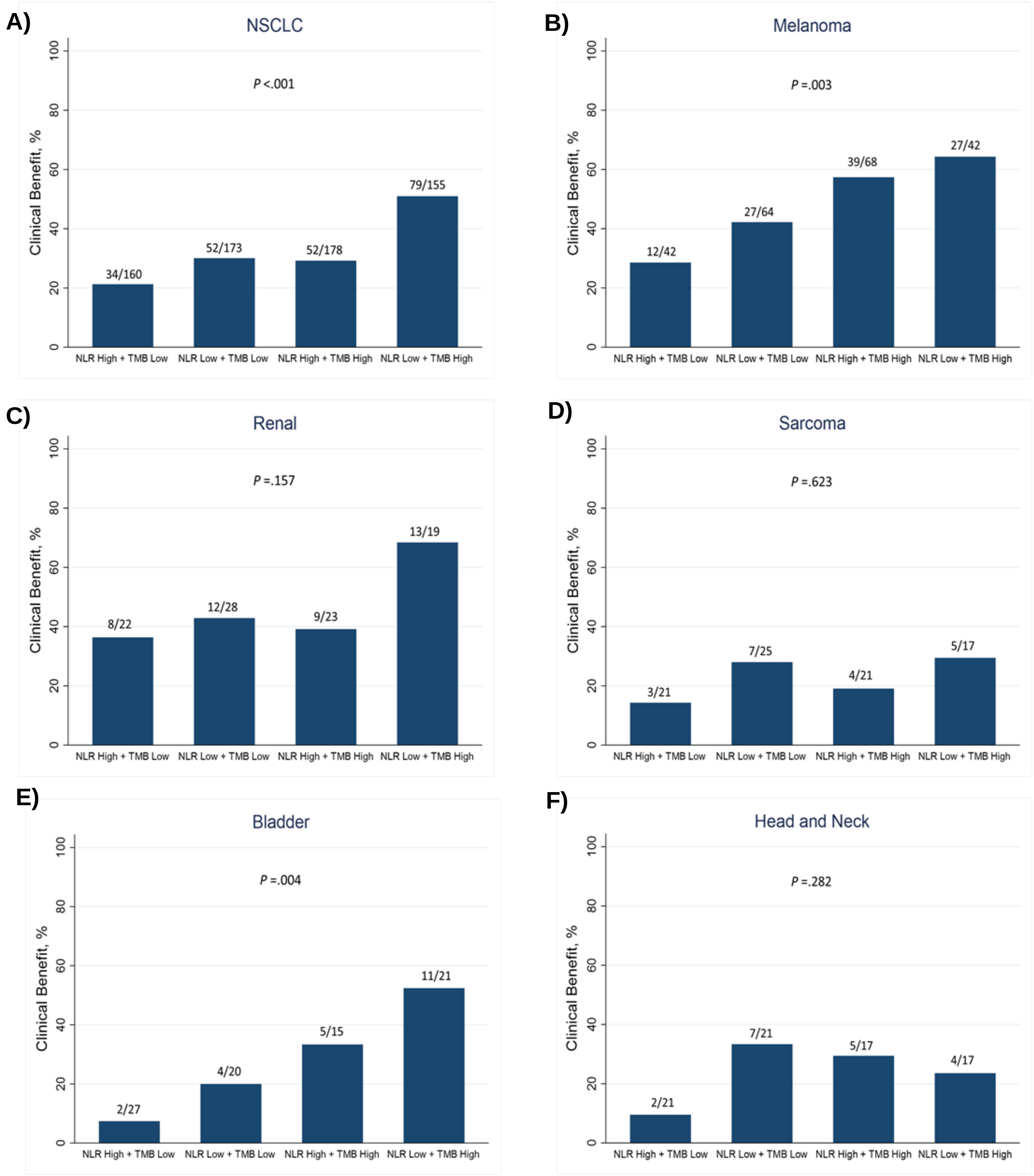


Clinical benefit based on a combined NLR and TMB variable stratified by cancer type for the main cancer types; A) non-small cell lung cancer (NSCLC), B) melanoma, C) renal, D) sarcoma, E) bladder, F) head and neck. We assigned patients into 4 categories based on NLR low/high and TMB low/high using median within cancer type as cutoff for both variables; P values according to Pearson´s chi-square test or Fisher´s exact test when appropriate.

## Supplementary Table 1. Characteristics of patients in the study

| Characteristic | No. patients (%) |
|---|---|
| Sex | |
| Female | 788 (46.0) |
| Male | 926 (54.0) |
| Age, median, years (IQR) | 64 (55-71) |
| Cancer type | |
| NSCLC | 666 (38.9) |
| Melanoma | 216 (12.6) |
| Renal | 92 (5.4) |
| Sarcoma | 84 (4.9) |
| Bladder | 83 (4.8) |
| Head and Neck | 76 (4.4) |
| Gastric | 71 (4.1) |
| Endometrial | 70 (4.1) |
| Colorectal | 68 (4.0) |
| Hepatobiliary | 57 (3.3) |
| SCLC | 54 (3.2) |
| Esophageal | 49 (2.9) |
| Pancreatic | 36 (2.1) |
| Mesothelioma | 35 (2.0) |
| Ovarian | 30 (1.8) |
| Breast | 27 (1.6) |
| Performance status | |
| ECOG 0 | 589 (34.4) |
| ECOG 1 | 860 (50.2) |
| ECOG ≥2 | 138 (8.1) |
| Unknown | 127 (7.4) |
| Drug class | |
| PD-1/PD-L1 | 1422 (83.0) |
| CTLA-4 | 7 (0.4) |
| Combo | 285 (16.6) |
| ICI line of treatment | |
| First line | 522 (30.5) |
| Subsequent line | 1192 (69.5) |
| Stage | |
| I-III | 105 (6.1) |
| IV | 1577 (92.0) |
| Unknown | 32 (1.9) |
| Year of treatment | |
| 2015-2016 | 448 (26.1) |
| 2017-2018 | 1266 (73.9) |
| MSI | |
| Stable | 1307 (76.3) |
| Indeterminate | 51 (3.0) |
| Unstable | 57 (3.3) |
| Unknown | 299 (17.4) |

Abbreviations: NSCLC, Non-small cell lung cancer; SCLC, small cell lung cancer; ECOG, Eastern Cooperative Oncology Group; PD-1, programmed cell death 1; PD-L1, programmed cell death ligand 1; CTLA-4, cytotoxic T-lymphocyte antigen 4; Combo, combination of anti-PD-1/PD-L1 and anti-CTLA-4; ICI, immune checkpoint inhibitor; MSI, microsatellite instability

## Supplementary Table 2. Characteristics of patients in the validation cohort (n = 323)

| Characteristic | No. patients (%) |
|---|---|
| Sex | |
| Female | 95 (29.4) |
| Male | 228 (70.6) |
| Age, median, years (IQR) | 60 (52-68) |
| Cancer type | |
| Renal | 136 (42.1) |
| CNS | 75 (23.2) |
| Head and Neck | 55 (17.0) |
| Unknown primary | 29 (9.0) |
| Bladder | 28 (8.7) |
| Performance status | |
| ECOG 0 | 142 (44.0) |
| ECOG 1 | 144 (44.6) |
| ECOG ≥2 | 26 (8.1) |
| Unknown | 11 (3.4) |
| Drug class | |
| PD-1/PD-L1 | 292 (90.4) |
| Combo | 31 (9.6) |
| ICI line of treatment | |
| First line | 61 (18.9) |
| Subsequent line | 262 (81.1) |
| Stage | |
| I-III | 8 (2.5) |
| IV | 240 (74.3) |
| Non-applicable | 75 (23.2) |
| Year of treatment | |
| 2014-2016 | 192 (59.4) |
| 2017-2019 | 131 (40.6) |
| MSI | |
| Stable | 267 (82.7) |
| Indeterminate | 6 (1.9) |
| Unstable | 3 (0.9) |
| Unknown | 47 (14.6) |

Abbreviations: CNS, Central nervous system; ECOG, Eastern Cooperative Oncology Group; PD-1, programmed cell death 1; PD-L1, programmed cell death ligand 1; Combo, combination of anti-PD-1/PD-L1 and anti-CTLA-4; CTLA-4, cytotoxic T-lymphocyte antigen 4; ICI, immune checkpoint inhibitor; MSI, microsatellite instability

**Supplementary Table 3. Factors associated with clinical benefit from immune checkpoint inhibitors (ICI) in the validation cohort (n = 323). Multivariable analysis including neutrophil-to-lymphocyte ratio (NLR) and tumor mutational burden (TMB) as continuous variables**

| | Univariate analysis | | Multivariable analysis | |
|---|---|---|---|---|
| | OR (95% CI) | P | OR (95% CI) | P |
| NLR | 0.868 (0.808-0.932) | <.001 | 0.920 (0.851-0.994) | .035 |
| TMB, Mutations/Mb | 1.023 (0.987-1.061) | .21 | 1.069 (1.021-1.120) | .004 |
| Age, y | 1.016 (0.998-1.033) | .08 | | |
| Sex | | | | |
| Female | Ref | | Ref | |
| Male | 1.709 (1.023-2.857) | .041 | 1.447 (0.805-2.599) | .22 |
| Cancer type | | | | |
| Renal | Ref | | Ref | |
| CNS | 0.265 (0.141-0.496) | <.001 | 0.276 (0.138-0.550) | <.001 |
| Head and Neck | 0.314 (0.159-0.622) | .001 | 0.505 (0.233-1.097) | .08 |
| Unknown primary | 0.134 (0.044-0.406) | <.001 | 0.188 (0.052-0.678) | .011 |
| Bladder | 0.628 (0.276-1.428) | .27 | 0.526 (0.203-1.363) | .19 |
| Performance status | | | | |
| ECOG 0 | Ref | | Ref | |
| ECOG 1 | 0.471 (0.292-0.761) | .002 | 0.674 (0.394-1.152) | .15 |
| ECOG ≥2 | 0.081 (0.018-0.356) | .001 | 0.144 (0.026-0.795) | .026 |
| Drug class | | | | |
| PD-1/PD-L1 | Ref | | | |
| Combo | 1.030 (0.481-2.203) | .08 | | |
| ICI line of treatment | | | | |
| First line | Ref | | Ref | |
| Subsequent line | 0.409 (0.232-0.720) | .002 | 0.477 (0.247-0.919) | .027 |
| Stage | | | | |
| I-III | Ref | | | |
| IV | 2.255 (0.446-11.404) | .33 | | |
| Year of treatment | | | | |
| 2014-2016 | Ref | | | |
| 2017-2019 | 0.854 (0.539-1.352) | .50 | | |

Odds ratios (ORs) were calculated with logistic regression.

Abbreviations: OR, Odds ratio; CI, confidence interval; Mb, megabase; CNS, central nervous system; ECOG, Eastern Cooperative Oncology Group; PD-1, programmed cell death 1; PD-L1, programmed cell death ligand 1; Combo, combination of anti-PD-1/PD-L1 and anti-CTLA-4; CTLA-4, cytotoxic T-lymphocyte antigen 4

**Supplementary Table 4. Factors associated with clinical benefit from immune checkpoint inhibitors (ICI). Multivariable analysis including neutrophil-to-lymphocyte ratio (NLR) and tumor mutational burden (TMB) as continuous variables**

| | Univariate analysis | | Multivariable analysis | |
|---|---|---|---|---|
| | **OR (95% CI)** | **P** | **OR (95% CI)** | **P** |
| NLR | 0.927 (0.904-0.950) | <.001 | 0.933 (0.906-0.961) | <.001 |
| TMB, Mutations/Mb | 1.030 (1.022-1.038) | <.001 | 1.031 (1.021-1.041) | <.001 |
| Age, y | 1.014 (1.006-1.022) | <.001 | 1.012 (1.002-1.022) | .022 |
| Sex | | | | |
| Female | Ref | | | |
| Male | 1.146 (0.935-1.405) | .19 | | |
| Cancer type | | | | |
| Melanoma | Ref | | Ref | |
| NSCLC | 0.511 (0.374-0.698) | <.001 | 1.503 (0.986-2.293) | .058 |
| Renal | 0.888 (0.544-1.449) | .63 | 3.249 (1.769-5.967) | <.001 |
| Sarcoma | 0.309 (0.174-0.550) | <.001 | 1.505 (0.724-3.128) | .27 |
| Bladder | 0.381 (0.219-0.665) | .001 | 1.019 (0.530-1.959) | .96 |
| Head and Neck | 0.328 (0.181-0.593) | <.001 | 2.009 (0.987-4.087) | .054 |
| Gastric | 0.611 (0.352-1.060) | .08 | 2.129 (1.101-4.120) | .025 |
| Endometrial | 0.664 (0.383-1.151) | .14 | 1.980 (1.012-3.876) | .046 |
| Colorectal | 0.352 (0.191-0.649) | .001 | 0.826 (0.373-1.826) | .64 |
| Hepatobiliary | 0.312 (0.159-0.613) | .001 | 1.197 (0.539-2.659) | .66 |
| SCLC | 0.270 (0.132-0.552) | <.001 | 1.165 (0.506-2.679) | .72 |
| Esophageal | 0.670 (0.355-1.262) | .22 | 2.815 (1.341-5.910) | .006 |
| Pancreatic | 0.030 (0.004-0.224) | .001 | 0.160 (0.021-1.232) | .08 |
| Mesothelioma | 0.313 (0.136-0.720) | .006 | 1.503 (0.589-3.833) | .39 |
| Ovarian | 0.211 (0.078-0.573) | .002 | 0.913 (0.307-2.715) | .87 |
| Breast | 0.184 (0.062-0.549) | .002 | 0.798 (0.215-2.963) | .74 |
| Performance status | | | | |
| ECOG 0 | Ref | | Ref | |
| ECOG 1 | 0.519 (0.416-0.647) | <.001 | 0.653 (0.504-0.847) | .001 |
| ECOG ≥2 | 0.203 (0.121-0.343) | <.001 | 0.244 (0.136-0.440) | <.001 |
| Drug class | | | | |
| PD-1/PD-L1 | Ref | | | |
| CTLA-4 | 0.362 (0.043-3.019) | .35 | | |
| Combo | 1.268 (0.973-1.654) | .08 | | |
| ICI line of treatment | | | | |
| First line | Ref | | Ref | |
| Subsequent line | 0.296 (0.238-0.367) | <.001 | 0.341 (0.256-0.456) | <.001 |
| Stage | | | | |
| I-III | Ref | | Ref | |
| IV | 0.480 (0.323-0.714) | <.001 | 0.687 (0.429-1.101) | .12 |
| Year of treatment | | | | |
| 2015-2016 | Ref | | Ref | |
| 2017-2018 | 0.792 (0.631-0.993) | .043 | 0.776 (0.592-1.019) | .07 |

Odds ratios (ORs) were calculated with logistic regression.

Abbreviations: OR, Odds ratio; CI, confidence interval; Mb, megabase; NSCLC, non-small cell lung cancer; SCLC, small cell lung cancer; ECOG, Eastern Cooperative Oncology Group; PD-1, programmed cell death 1; PD-L1, programmed cell death ligand 1; CTLA-4, cytotoxic T-lymphocyte antigen 4; Combo, combination of anti-PD-1/PD-L1 and anti-CTLA-4

**Supplementary Table 5. Factors associated with clinical benefit from immune checkpoint inhibitors (ICI). Multivariable analysis including neutrophil-to-lymphocyte ratio (NLR) and tumor mutational burden (TMB) as percentile within cancer type**

| | Univariate analysis | | Multivariable analysis | |
|---|---|---|---|---|
| | **OR (95% CI)** | **P** | **OR (95% CI)** | **P** |
| NLR | | | | |
| Bottom 80% | Ref | | Ref | |
| Top 20% | 0.501 (0.379-0.663) | <.001 | 0.554 (0.400-0.766) | <.001 |
| TMB | | | | |
| Bottom 80% | Ref | | Ref | |
| Top 20% | 2.588 (2.027-3.304) | <.001 | 3.155 (2.368-4.203) | <.001 |
| Age, y | 1.014 (1.006-1.022) | <.001 | 1.014 (1.004-1.025) | .005 |
| Sex | | | | |
| Female | Ref | | | |
| Male | 1.146 (0.935-1.405) | .19 | | |
| Cancer type | | | | |
| Melanoma | Ref | | Ref | |
| NSCLC | 0.511 (0.374-0.698) | <.001 | 0.963 (0.641-1.446) | .86 |
| Renal | 0.888 (0.544-1.449) | .63 | 2.041 (1.121-3.716) | .020 |
| Sarcoma | 0.309 (0.174-0.550) | <.001 | 0.934 (0.454-1.919) | .85 |
| Bladder | 0.381 (0.219-0.665) | .001 | 0.756 (0.394-1.452) | .40 |
| Head and Neck | 0.328 (0.181-0.593) | <.001 | 1.019 (0.509-2.042) | .96 |
| Gastric | 0.611 (0.352-1.060) | .08 | 1.438 (0.746-2.771) | .28 |
| Endometrial | 0.664 (0.383-1.151) | .14 | 1.768 (0.911-3.429) | .09 |
| Colorectal | 0.352 (0.191-0.649) | .001 | 1.084 (0.522-2.250) | .83 |
| Hepatobiliary | 0.312 (0.159-0.613) | .001 | 0.820 (0.373-1.803) | .62 |
| SCLC | 0.270 (0.132-0.552) | <.001 | 0.710 (0.305-1.650) | .43 |
| Esophageal | 0.670 (0.355-1.262) | .22 | 2.067 (0.989-4.321) | .053 |
| Pancreatic | 0.030 (0.004-0.224) | .001 | 0.090 (0.012-0.699) | .021 |
| Mesothelioma | 0.313 (0.136-0.720) | .006 | 0.813 (0.323-2.045) | .66 |
| Ovarian | 0.211 (0.078-0.573) | .002 | 0.597 (0.200-1.783) | .36 |
| Breast | 0.184 (0.062-0.549) | .002 | 0.542 (0.143-2.055) | .37 |
| Performance status | | | | |
| ECOG 0 | Ref | | Ref | |
| ECOG 1 | 0.519 (0.416-0.647) | <.001 | 0.597 (0.461-0.772) | <.001 |
| ECOG ≥2 | 0.203 (0.121-0.343) | <.001 | 0.208 (0.117-0.370) | <.001 |
| Drug class | | | | |
| PD-1/PD-L1 | Ref | | | |
| CTLA-4 | 0.362 (0.043-3.019) | .35 | | |
| Combo | 1.268 (0.973-1.654) | .08 | | |
| ICI line of treatment | | | | |
| First line | Ref | | Ref | |
| Subsequent line | 0.296 (0.238-0.367) | <.001 | 0.328 (0.245-0.438) | <.001 |
| Stage | | | | |
| I-III | Ref | | Ref | |
| IV | 0.480 (0.323-0.714) | <.001 | 0.672 (0.421-1.072) | .10 |
| Year of treatment | | | | |
| 2015-2016 | Ref | | Ref | |
| 2017-2018 | 0.792 (0.631-0.993) | .043 | 0.756 (0.576-0.992) | .04 |

Odds ratios (ORs) were calculated with logistic regression.

Abbreviations: OR, Odds ratio; CI, confidence interval; NSCLC, non-small cell lung cancer; SCLC, small cell lung cancer; ECOG, Eastern Cooperative Oncology Group; PD-1, programmed cell death 1; PD-L1, programmed cell death ligand 1; CTLA-4, cytotoxic T-lymphocyte antigen 4; Combo, combination of anti-PD-1/PD-L1 and anti-CTLA-4

**Supplementary Table 6. Factors associated with overall survival from treatment with immune checkpoint inhibitors (ICI)**

| | Univariate analysis | | Multivariable analysis | |
|---|---|---|---|---|
| | HR (95% CI) | P | HR (95% CI) | P |
| NLR + TMB[a] | | | | |
| NLR high + TMB low | Ref | | Ref | |
| NLR low + TMB low | 0.493 (0.419-0.582) | <.001 | 0.586 (0.491-0.699) | <.001 |
| NLR high + TMB high | 0.661 (0.562-0.777) | <.001 | 0.726 (0.613-0.861) | <.001 |
| NLR low + TMB high | 0.387 (0.322-0.466) | <.001 | 0.416 (0.342-0.506) | <.001 |
| Age, y | 1.004 (0.999-1.009) | .10 | | |
| Sex | | | | |
| Female | Ref | | | |
| Male | 0.940 (0.831-1.062) | .32 | | |
| Cancer type | | | | |
| Melanoma | Ref | | Ref | |
| NSCLC | 2.227 (1.766-2.808) | <.001 | 1.326 (0.994-1.768) | .055 |
| Renal | 1.208 (0.841-1.737) | .31 | 0.845 (0.565-1.264) | .41 |
| Sarcoma | 2.346 (1.650-3.335) | <.001 | 1.461 (0.950-2.246) | .08 |
| Bladder | 2.303 (1.645-3.225) | <.001 | 1.308 (0.895-1.909) | .17 |
| Head and Neck | 2.240 (1.573-3.190) | <.001 | 1.066 (0.709-1.602) | .76 |
| Gastric | 2.970 (2.094-4.212) | <.001 | 2.048 (1.400-2.997) | <.001 |
| Endometrial | 1.578 (1.064-2.340) | .023 | 0.896 (0.575-1.397) | .63 |
| Colorectal | 2.458 (1.699-3.557) | <.001 | 1.536 (1.009-2.340) | .045 |
| Hepatobiliary | 2.355 (1.583-3.504) | <.001 | 1.272 (0.814-1.988) | .29 |
| SCLC | 3.782 (2.620-5.459) | <.001 | 2.155 (1.414-3.283) | <.001 |
| Esophageal | 2.917 (1.934-4.401) | <.001 | 1.779 (1.140-2.775) | .011 |
| Pancreatic | 4.214 (2.711-6.549) | <.001 | 2.267 (1.399-3.675) | .001 |
| Mesothelioma | 2.828 (1.810-4.418) | <.001 | 1.488 (0.921-2.405) | .11 |
| Ovarian | 2.855 (1.787-4.563) | <.001 | 1.678 (1.009-2.790) | .046 |
| Breast | 2.823 (1.698-4.695) | <.001 | 1.512 (0.839-2.726) | .17 |
| Performance status | | | | |
| ECOG 0 | Ref | | Ref | |
| ECOG 1 | 2.032 (1.756-2.350) | <.001 | 1.677 (1.438-1.954) | <.001 |
| ECOG ≥2 | 4.655 (3.737-5.798) | <.001 | 4.040 (3.208-5.088) | <.001 |
| Drug class | | | | |
| PD-1/PD-L1 | Ref | | Ref | |
| CTLA-4 | 1.351 (0.605-3.016) | .46 | 1.580 (0.626-3.989) | .33 |
| Combo | 0.764 (0.645-0.906) | .002 | 0.910 (0.747-1.110) | .35 |
| ICI line of treatment | | | | |
| First line | Ref | | Ref | |
| Subsequent line | 2.185 (1.886-2.533) | <.001 | 1.879 (1.565-2.255) | <.001 |
| Stage | | | | |
| I-III | Ref | | Ref | |
| IV | 2.456 (1.761-3.426) | <.001 | 1.922 (1.356-2.724) | <.001 |
| Year of treatment | | | | |
| 2015-2016 | Ref | | | |
| 2017-2018 | 1.092 (0.950-1.255) | .22 | | |

[a]Neutrophil-to-lymphocyte ratio (NLR) and tumor mutational burden (TMB) were stratified into high and low using median within cancer type as cutoff. Hazard ratios (HRs) were calculated with Cox proportional hazard regression.

Abbreviations: HR, Hazard ratio; CI, confidence interval; NSCLC, non-small cell lung cancer; SCLC, small cell lung cancer; ECOG, Eastern Cooperative Oncology Group; PD-1, programmed cell death 1; PD-L1, programmed cell death ligand 1; CTLA-4, cytotoxic T-lymphocyte antigen 4; Combo, combination of anti-PD-1/PD-L1 and anti-CTLA-4

**Supplementary Table 7. Factors associated with progression-free survival from treatment with immune checkpoint inhibitors (ICI)**

| | Univariate analysis | | Multivariable analysis | |
|---|---|---|---|---|
| | HR (95% CI) | P | HR (95% CI) | P |
| NLR + TMB[a] | | | | |
| NLR high + TMB low | Ref | | Ref | |
| NLR low + TMB low | 0.687 (0.597-0.789) | <.001 | 0.745 (0.641-0.867) | <.001 |
| NLR high + TMB high | 0.663 (0.574-0.765) | <.001 | 0.686 (0.590-0.798) | <.001 |
| NLR low + TMB high | 0.475 (0.408-0.554) | <.001 | 0.504 (0.428-0.594) | <.001 |
| Age, y | 0.994 (0.990-0.998) | .004 | 0.994 (0.989-0.998) | .006 |
| Sex | | | | |
| Female | Ref | | | |
| Male | 0.947 (0.854-1.050) | .30 | | |
| Cancer type | | | | |
| Melanoma | Ref | | Ref | |
| NSCLC | 1.659 (1.383-1.991) | <.001 | 1.140 (0.912-1.426) | .25 |
| Renal | 1.046 (0.782-1.399) | .76 | 0.713 (0.518-0.983) | .039 |
| Sarcoma | 2.257 (1.715-2.971) | <.001 | 1.353 (0.972-1.883) | .07 |
| Bladder | 1.619 (1.212-2.162) | .001 | 1.111 (0.809-1.527) | .52 |
| Head and Neck | 1.986 (1.489-2.650) | <.001 | 1.075 (0.775-1.490) | .67 |
| Gastric | 1.959 (1.464-2.622) | <.001 | 1.359 (0.994-1.859) | .055 |
| Endometrial | 1.431 (1.055-1.941) | .021 | 0.900 (0.639-1.267) | .55 |
| Colorectal | 1.834 (1.350-2.492) | <.001 | 1.182 (0.835-1.672) | .35 |
| Hepatobiliary | 1.783 (1.293-2.459) | <.001 | 1.052 (0.738-1.499) | .78 |
| SCLC | 2.728 (1.985-3.749) | <.001 | 1.817 (1.269-2.601) | .001 |
| Esophageal | 1.591 (1.117-2.265) | .010 | 1.038 (0.714-1.510) | .85 |
| Pancreatic | 4.147 (2.872-5.988) | <.001 | 2.529 (1.704-3.754) | <.001 |
| Mesothelioma | 1.979 (1.349-2.903) | <.001 | 1.136 (0.754-1.712) | .54 |
| Ovarian | 1.882 (1.239-2.858) | .003 | 1.162 (0.746-1.809) | .51 |
| Breast | 2.043 (1.325-3.148) | .001 | 1.156 (0.718-1.862) | .55 |
| Performance status | | | | |
| ECOG 0 | Ref | | Ref | |
| ECOG 1 | 1.523 (1.355-1.711) | <.001 | 1.363 (1.203-1.545) | <.001 |
| ECOG ≥2 | 2.764 (2.271-3.364) | <.001 | 2.536 (3.208-3.122) | <.001 |
| Drug class | | | | |
| PD-1/PD-L1 | Ref | | | |
| CTLA-4 | 1.295 (0.605-2.722) | .50 | | |
| Combo | 0.933 (0.811-1.074) | .33 | | |
| ICI line of treatment | | | | |
| First line | Ref | | Ref | |
| Subsequent line | 1.883 (1.672-2.121) | <.001 | 1.703 (1.467-1.977) | <.001 |
| Stage | | | | |
| I-III | Ref | | Ref | |
| IV | 1.760 (1.383-2.238) | <.001 | 1.555 (1.206-2.006) | .001 |
| Year of treatment | | | | |
| 2015-2016 | Ref | | | |
| 2017-2018 | 1.096 (0.974-1.234) | .13 | | |

[a]Neutrophil-to-lymphocyte ratio (NLR) and tumor mutational burden (TMB) were stratified into high and low using median within cancer type as cutoff. Hazard ratios (HRs) were calculated with Cox proportional hazard regression.

Abbreviations: HR, Hazard ratio; CI, confidence interval; NSCLC, non-small cell lung cancer; SCLC, small cell lung cancer; ECOG, Eastern Cooperative Oncology Group; PD-1, programmed cell death 1; PD-L1, programmed cell death ligand 1; CTLA-4, cytotoxic T-lymphocyte antigen 4; Combo, combination of anti-PD-1/PD-L1 and anti-CTLA-4

**Supplementary Table 8. Factors associated with response from treatment with immune checkpoint inhibitors (ICI)**

| | Univariate analysis | | Multivariable analysis | |
|---|---|---|---|---|
| | OR (95% CI) | P | OR (95% CI) | P |
| NLR + TMB[a] | | | | |
| NLR high + TMB low | Ref | | Ref | |
| NLR low + TMB low | 1.578 (1.133-2.198) | .007 | 1.358 (0.933-1.977) | .11 |
| NLR high + TMB high | 1.996 (1.437-2.771) | <.001 | 1.902 (1.318-2.744) | .001 |
| NLR low + TMB high | 3.075 (2.216-4.267) | <.001 | 2.786 (1.923-4.037) | <.001 |
| Age, y | 1.012 (1.004-1.021) | .004 | 1.014 (1.003-1.024) | .012 |
| Sex | | | | |
| Female | Ref | | | |
| Male | 1.171 (0.944-1.453) | .15 | | |
| Cancer type | | | | |
| Melanoma | Ref | | Ref | |
| NSCLC | 0.414 (0.300-0.570) | <.001 | 0.732 (0.483-1.111) | .14 |
| Renal | 0.825 (0.503-1.355) | .45 | 1.653 (0.903-3.024) | .10 |
| Sarcoma | 0.289 (0.157-0.530) | <.001 | 0.734 (0.346-1.559) | .42 |
| Bladder | 0.249 (0.132-0.469) | <.001 | 0.472 (0.230-0.969) | .041 |
| Head and Neck | 0.353 (0.194-0.646) | .001 | 1.112 (0.547-2.260) | .77 |
| Gastric | 0.515 (0.290-0.916) | .024 | 1.085 (0.558-2.109) | .81 |
| Endometrial | 0.526 (0.295-0.936) | .029 | 1.339 (0.675-2.659) | .40 |
| Colorectal | 0.318 (0.167-0.607) | .001 | 0.872 (0.405-1.876) | .73 |
| Hepatobiliary | 0.200 (0.091-0.443) | <.001 | 0.594 (0.247-1.430) | .25 |
| SCLC | 0.314 (0.154-0.641) | .001 | 0.805 (0.349-1.855) | .61 |
| Esophageal | 0.652 (0.341-1.244) | .19 | 1.766 (0.844-3.698) | .13 |
| Pancreatic | 0.035 (0.005-0.260) | .001 | 0.101 (0.013-0.781) | .028 |
| Mesothelioma | 0.254 (0.101-0.636) | .003 | 0.694 (0.258-1.867) | .47 |
| Ovarian | 0.245 (0.091-0.665) | .006 | 0.711 (0.241-2.102) | .54 |
| Breast | 0.213 (0.071-0.638) | .006 | 0.675 (0.178-2.556) | .56 |
| Performance status | | | | |
| ECOG 0 | Ref | | Ref | |
| ECOG 1 | 0.501 (0.397-0.632) | <.001 | 0.596 (0.455-0.780) | <.001 |
| ECOG ≥2 | 0.232 (0.134-0.400) | <.001 | 0.257 (0.143-0.463) | <.001 |
| Drug class | | | | |
| PD-1/PD-L1 | Ref | | Ref | |
| CTLA-4 | - | - | - | - |
| Combo | 1.552 (1.182-2.037) | .002 | 1.082 (0.765-1.530) | .66 |
| ICI line of treatment | | | | |
| First line | Ref | | Ref | |
| Subsequent line | 0.287 (0.229-0.360) | <.001 | 0.334 (0.247-0.452) | <.001 |
| Stage | | | | |
| I-III | Ref | | Ref | |
| IV | 0.621 (0.411-0.940) | .024 | 1.015 (0.619-1.663) | .95 |
| Year of treatment | | | | |
| 2015-2016 | Ref | | Ref | |
| 2017-2018 | 0.746 (0.588-0.945) | .015 | 0.719 (0.541-0.959) | .023 |

[a]Neutrophil-to-lymphocyte ratio (NLR) and tumor mutational burden (TMB) were stratified into high and low using median within cancer type as cutoff. Odds ratios (ORs) were calculated with logistic regression.

Abbreviations: OR, Odds ratio; CI, confidence interval; NSCLC, non-small cell lung cancer; SCLC, small cell lung cancer; ECOG, Eastern Cooperative Oncology Group; PD-1, programmed cell death 1; PD-L1, programmed cell death ligand 1; CTLA-4, cytotoxic T-lymphocyte antigen 4; Combo, combination of anti-PD-1/PD-L1 and anti-CTLA-4

**Supplementary Table 9. Factors associated with clinical benefit from treatment with immune checkpoint inhibitors (ICI) in stage III-IV tumors (n = 1,671)**

| | Univariate analysis | | Multivariable analysis | |
|---|---|---|---|---|
| | **OR (95% CI)** | **P** | **OR (95% CI)** | **P** |
| NLR + TMB[a] | | | | |
| NLR high + TMB low | Ref | | Ref | |
| NLR low + TMB low | 1.649 (1.206-2.255) | .002 | 1.516 (1.065-2.157) | .021 |
| NLR high + TMB high | 1.946 (1.426-2.656) | <.001 | 1.888 (1.333-2.674) | <.001 |
| NLR low + TMB high | 3.527 (2.579-4.824) | <.001 | 3.263 (2.289-4.652) | <.001 |
| Age, y | 1.015 (1.007-1.023) | <.001 | 1.014 (1.004-1.024) | .006 |
| Sex | | | | |
| Female | Ref | | | |
| Male | 1.147 (0.933-1.410) | .19 | | |
| Cancer type | | | | |
| Melanoma | Ref | | Ref | |
| NSCLC | 0.490 (0.355-0.678) | <.001 | 0.995 (0.665-1.490) | .98 |
| Renal | 0.849 (0.516-1.395) | .52 | 2.059 (1.135-3.734) | .017 |
| Sarcoma | 0.307 (0.166-0.565) | <.001 | 0.901 (0.441-1.841) | .78 |
| Bladder | 0.377 (0.214-0.662) | .001 | 0.836 (0.435-1.606) | .59 |
| Head and Neck | 0.314 (0.172-0.571) | <.001 | 1.079 (0.541-2.152) | .83 |
| Gastric | 0.584 (0.334-1.020) | .059 | 1.486 (0.778-2.840) | .23 |
| Endometrial | 0.634 (0.363-1.107) | .11 | 1.831 (0.949-3.531) | .07 |
| Colorectal | 0.317 (0.169-0.594) | <.001 | 1.024 (0.487-2.154) | .95 |
| Hepatobiliary | 0.253 (0.123-0.518) | <.001 | 0.711 (0.317-1.594) | .41 |
| SCLC | 0.258 (0.126-0.531) | <.001 | 0.759 (0.331-1.738) | .51 |
| Esophageal | 0.640 (0.338-1.213) | .17 | 1.937 (0.934-4.017) | .08 |
| Pancreatic | 0.029 (0.004-0.215) | .001 | 0.092 (0.012-0.707) | .022 |
| Mesothelioma | 0.283 (0.117-0.685) | .005 | 0.829 (0.317-2.166) | .70 |
| Ovarian | 0.202 (0.074-0.549) | .002 | 0.655 (0.222-1.931) | .44 |
| Breast | 0.132 (0.038-0.453) | .001 | 0.571 (0.154-2.120) | .40 |
| Performance status | | | | |
| ECOG 0 | Ref | | Ref | |
| ECOG 1 | 0.515 (0.411-0.644) | <.001 | 0.621 (0.480-0.805) | <.001 |
| ECOG ≥2 | 0.204 (0.121-0.344) | <.001 | 0.237 (0.135-0.415) | <.001 |
| Drug class | | | | |
| PD-1/PD-L1 | Ref | | | |
| CTLA-4 | 0.436 (0.051-3.739) | .45 | | |
| Combo | 1.261 (0.960-1.655) | .10 | | |
| ICI line of treatment | | | | |
| First line | Ref | | Ref | |
| Subsequent line | 0.283 (0.226-0.353) | <.001 | 0.323 (0.241-0.431) | <.001 |
| Stage | | | | |
| III | Ref | | Ref | |
| IV | 0.453 (0.299-0.689) | <.001 | 0.650 (0.400-1.057) | .08 |
| Year of treatment | | | | |
| 2015-2016 | Ref | | Ref | |
| 2017-2018 | 0.792 (0.629-0.997) | .047 | 0.765 (0.583-1.003) | .052 |

[a]Neutrophil-to-lymphocyte ratio (NLR) and tumor mutational burden (TMB) were stratified into high and low using median within cancer type as cutoff. Odds ratios (ORs) were calculated with logistic regression.

Abbreviations: OR, Odds ratio; CI, confidence interval; NSCLC, non-small cell lung cancer; SCLC, small cell lung cancer; ECOG, Eastern Cooperative Oncology Group; PD-1, programmed cell death 1; PD-L1, programmed cell death ligand 1; CTLA-4, cytotoxic T-lymphocyte antigen 4; Combo, combination of anti-PD-1/PD-L1 and anti-CTLA-4

**Supplementary Table 10. Factors associated with clinical benefit from treatment with immune checkpoint inhibitors (ICI) in microsatellite stable tumors (n = 1,358)**

| | Univariate analysis | | Multivariable analysis | |
|---|---|---|---|---|
| | OR (95% CI) | P | OR (95% CI) | P |
| NLR + TMB[a] | | | | |
| NLR high + TMB low | Ref | | Ref | |
| NLR low + TMB low | 1.492 (1.059-2.100) | .022 | 1.390 (0.935-2.065) | .10 |
| NLR high + TMB high | 1.909 (1.356-2.689) | <.001 | 1.914 (1.291-2.839) | .001 |
| NLR low + TMB high | 3.435 (2.431-4.853) | <.001 | 3.142 (2.098-4.706) | <.001 |
| Age, y | 1.012 (1.003-1.022) | .008 | 1.012 (1.000-1.024) | .047 |
| Sex | | | | |
| Female | Ref | | | |
| Male | 1.118 (0.888-1.408) | .34 | | |
| Cancer type | | | | |
| Melanoma | Ref | | Ref | |
| NSCLC | 0.479 (0.335-0.685) | <.001 | 0.912 (0.568-1.464) | .70 |
| Renal | 0.831 (0.486-1.419) | .50 | 1.907 (0.986-3.686) | .055 |
| Sarcoma | 0.298 (0.157-0.565) | <.001 | 0.845 (0.384-1.858) | .68 |
| Bladder | 0.282 (0.144-0.551) | <.001 | 0.511 (0.232-1.123) | .10 |
| Head and Neck | 0.292 (0.146-0.583) | <.001 | 0.851 (0.379-1.913) | .70 |
| Gastric | 0.633 (0.351-1.142) | .13 | 1.526 (0.756-3.080) | .24 |
| Endometrial | 0.463 (0.233-0.920) | .028 | 1.416 (0.637-3.151) | .39 |
| Colorectal | 0.096 (0.033-0.282) | <.001 | 0.290 (0.091-0.920) | .036 |
| Hepatobiliary | 0.219 (0.096-0.501) | <.001 | 0.614 (0.239-1.576) | .31 |
| SCLC | 0.254 (0.115-0.562) | .001 | 0.686 (0.270-1.744) | .43 |
| Esophageal | 0.576 (0.278-1.193) | .14 | 1.494 (0.648-3.445) | .35 |
| Pancreatic | 0.030 (0.004-0.224) | .001 | 0.084 (0.011-0.660) | .018 |
| Mesothelioma | 0.296 (0.113-0.776) | .013 | 0.769 (0.263-2.246) | .63 |
| Ovarian | 0.232 (0.075-0.721) | .012 | 0.755 (0.222-2.569) | .65 |
| Breast | 0.219 (0.071-0.677) | .008 | 0.632 (0.158-2.524) | .52 |
| Performance status | | | | |
| ECOG 0 | Ref | | Ref | |
| ECOG 1 | 0.485 (0.378-0.623) | <.001 | 0.580 (0.433-0.777) | <.001 |
| ECOG ≥2 | 0.135 (0.067-0.275) | <.001 | 0.148 (0.070-0.312) | <.001 |
| Drug class | | | | |
| PD-1/PD-L1 | Ref | | Ref | |
| CTLA-4 | 0.383 (0.046-3.195) | .38 | 0.388 (0.037-4.039) | .43 |
| Combo | 1.435 (1.061-1.941) | .019 | 1.112 (0.757-1.634) | .59 |
| ICI line of treatment | | | | |
| First line | Ref | | Ref | |
| Subsequent line | 0.277 (0.217-0.355) | <.001 | 0.325 (0.235-0.450) | <.001 |
| Stage | | | | |
| I-III | Ref | | Ref | |
| IV | 0.491 (0.308-0.782) | .003 | 0.653 (0.374-1.139) | .13 |
| Year of treatment | | | | |
| 2015-2016 | Ref | | | |
| 2017-2018 | 0.901 (0.692-1.175) | .44 | | |

[a]Neutrophil-to-lymphocyte ratio (NLR) and tumor mutational burden (TMB) were stratified into high and low using median within cancer type as cutoff. Odds ratios (ORs) were calculated with logistic regression.

Abbreviations: OR, Odds ratio; CI, confidence interval; NSCLC, non-small cell lung cancer; SCLC, small cell lung cancer; ECOG, Eastern Cooperative Oncology Group; PD-1, programmed cell death 1; PD-L1, programmed cell death ligand 1; CTLA-4, cytotoxic T-lymphocyte antigen 4; Combo, combination of anti-PD-1/PD-L1 and anti-CTLA-4
