# Supplementary material for: Pretreatment neutrophil-to-lymphocyte ratio and mutational burden as biomarkers of tumor response to immune checkpoint inhibitors
Source: Nat Commun. 2021 Feb 1;12:729. doi: 10.1038/s41467-021-20935-9 (PMC7851155; doi:10.1038/s41467-021-20935-9)
Supplement: Supplementary file 2 — Description of Additional Supplementary Files [file 41467_2021_20935_MOESM2_ESM.pdf]

Description of Additional Supplementary Files

File Name: Supplementary Data 1
Description: Complete NLR, TMB, and other clinical, pathological and genomic data for 1714 ICI-treated patients in the primary cohort and 323 ICI-treated patients in the validation cohort, and the genes on each version of the IMPACT panel.
