# Supplementary material for: Pretreatment neutrophil-to-lymphocyte ratio and mutational burden as biomarkers of tumor response to immune checkpoint inhibitors
Source: Nat Commun. 2021 Feb 1;12:729. doi: 10.1038/s41467-021-20935-9 (PMC7851155; doi:10.1038/s41467-021-20935-9)
Supplement: Supplementary file 4 — Reporting Summary [file 41467_2021_20935_MOESM4_ESM.pdf]

nature research

Corresponding author(s): Luc G.T. Morris and Venkatraman E. Seshan

Last updated by author(s): Nov 27, 2020

Reporting Summary

Nature Research wishes to improve the reproducibility of the work that we publish. This form provides structure for consistency and transparency in reporting. For further information on Nature Research policies, see Authors & Referees and the Editorial Policy Checklist .

Statistics

For all statistical analyses, confirm that the following items are present in the figure legend, table legend, main text, or Methods section.

n/a	Confirmed	
☐	☒	The exact sample size (*n*) for each experimental group/condition, given as a discrete number and unit of measurement
☐	☒	A statement on whether measurements were taken from distinct samples or whether the same sample was measured repeatedly
☐	☒	The statistical test(s) used AND whether they are one- or two-sided<br>*Only common tests should be described solely by name; describe more complex techniques in the Methods section.*
☐	☒	A description of all covariates tested
☐	☒	A description of any assumptions or corrections, such as tests of normality and adjustment for multiple comparisons
☐	☒	A full description of the statistical parameters including central tendency (e.g. means) or other basic estimates (e.g. regression coefficient) AND variation (e.g. standard deviation) or associated estimates of uncertainty (e.g. confidence intervals)
☐	☒	For null hypothesis testing, the test statistic (e.g. *F*, *t*, *r*) with confidence intervals, effect sizes, degrees of freedom and *P* value noted<br>*Give P values as exact values whenever suitable.*
☒	☐	For Bayesian analysis, information on the choice of priors and Markov chain Monte Carlo settings
☐	☒	For hierarchical and complex designs, identification of the appropriate level for tests and full reporting of outcomes
☐	☒	Estimates of effect sizes (e.g. Cohen's *d*, Pearson's *r*), indicating how they were calculated

Our web collection on statistics for biologists contains articles on many of the points above.

Software and code

Policy information about availability of computer code

Data collection	No software was used for data collection.
Data analysis	All statistical analyses were conducted using Stata (Stata Statistical Software: Release 16; StataCorp LLC).

For manuscripts utilizing custom algorithms or software that are central to the research but not yet described in published literature, software must be made available to editors/reviewers. We strongly encourage code deposition in a community repository (e.g. GitHub). See the Nature Research guidelines for submitting code & software for further information.

Data

Policy information about availability of data

All manuscripts must include a data availability statement. This statement should provide the following information, where applicable:

- Accession codes, unique identifiers, or web links for publicly available datasets
- A list of figures that have associated raw data
- A description of any restrictions on data availability

The de-identified clinical, NLR and TMB data used for the analyses in this study have been deposited in the Zenodo database under accession code DOI: 10.5281/zenodo.4293814 [https://zenodo.org/record/4293814#.X8ElGc1KjIU].

Field-specific reporting

Please select the one below that is the best fit for your research. If you are not sure, read the appropriate sections before making your selection.

☒ Life sciences ☐ Behavioural & social sciences ☐ Ecological, evolutionary & environmental sciences

For a reference copy of the document with all sections, see nature.com/documents/nr-reporting-summary-flat.pdf

Life sciences study design

All studies must disclose on these points even when the disclosure is negative.

Sample size

Patients initially selected for the study were all those with solid tumors diagnosed from 2015 through 2018 who received at least 1 dose of Immune Checkpoint Inhibitor at our center (n = 2,827). All tumors, along with DNA from peripheral blood, were genomically profiled using the MSK-IMPACT next-generation sequencing platform. After all the exclusions, the final cohort consisted of 1,714 patients with 16 cancer types.

After completion of all analyses and initial peer review of the manuscript, we obtained data for an independent cohort of 323 additional patients treated at our center, for validation of the NLR thresholds analyzed in the primary cohort. This cohort used identical inclusion criteria as the primary cohort, but extended the years of eligibility to patients treated between 2014 to 2019.

The sample size was based on all available patients and the detectable alternative then examined. Planning a study of 1,714 patients, assuming that we would compare patient NLR categories of the top 20% compared to the bottom 80% of patients, and assuming a median survival time of 18 months, this sample size would offer sufficient power to detect true hazard ratios of failure for control subjects relative to experimental subjects of 0.843 or 1.188 with power 0.8, and Type I error probability associated with this test of the null hypothesis that the experimental and control survival curves are equal is 0.05.

Data exclusions

We excluded patients with history of more than 1 cancer, those without a complete blood count within 30 days prior to the first dose of Immune Checkpoint Inhibitor, those enrolled in blinded trials, and cancer types with fewer than 25 cases. We excluded patients who received Immune Checkpoint Inhibitors in a neoadjuvant or adjuvant setting, and patients with unevaluable response (lost to follow-up without imaging after treatment start).

Replication

After initial analyses and peer review of the primary cohort of 1,714, an additional separate validation cohort of 323 patients was obtained by extending years of eligibility, in order to replicate the primary analyses.

Randomization

The study was a retrospective cohort study, no randomization was performed.

Blinding

The clinical records of the patients were manually reviewed to assess respone to therapy, progression-free survival and overall survival. The process was blinded to patients' Neutrophil-to-lymphocyte ratio and Tumor mutational burden values.

Reporting for specific materials, systems and methods

We require information from authors about some types of materials, experimental systems and methods used in many studies. Here, indicate whether each material, system or method listed is relevant to your study. If you are not sure if a list item applies to your research, read the appropriate section before selecting a response.

Materials & experimental systems

n/a	Involved in the study
☒	☐ Antibodies
☒	☐ Eukaryotic cell lines
☒	☐ Palaeontology
☒	☐ Animals and other organisms
☐	☒ Human research participants
☒	☐ Clinical data

Methods

n/a	Involved in the study
☒	☐ ChIP-seq
☒	☐ Flow cytometry
☒	☐ MRI-based neuroimaging

Human research participants

Policy information about studies involving human research participants

Population characteristics	To assess the predictive value of pre-treatment NLR, we analyzed data for 1,714 patients with response and survival outcomes after ICI treatment. For the initial cohort, median age was 64 years (IQR, 55-71); 926 patients (54%) were male.<br><br>For the validation cohort (n = 323), median age was 60 years (IQR, 52-68); 228 patients (71%) were male.
Recruitment	Based on a dataline request, patients with solid tumors who were first diagnosed during 2015 through 2018, whose tumors underwent next-generation DNA sequencing with MSK-IMPACT, and who received subsequent cancer therapy at Memorial Sloan Kettering Cancer Center were recruited.<br><br>After completion of all analyses and initial peer review of the manuscript, we obtained data for an independent cohort of 323 additional patients treated at our center extending the years of eligibility to patients treated between 2014 to 2019.<br><br>The entire cohort of all available patients was included, and there were no additional selection or exclusions.
Ethics oversight	Memorial Sloan Kettering Cancer Center Institutional Review Board

Note that full information on the approval of the study protocol must also be provided in the manuscript.
